# The Typology of V2 and the Distribution of Pleonastic die in the Ghent Dialect

**DOI:** 10.3389/fpsyg.2018.01342

**Published:** 2018-08-29

**Authors:** Karen De Clercq, Liliane Haegeman

**Affiliations:** ^1^DIALING, Department of Linguistics, Ghent University, Ghent, Belgium; ^2^FWO, Department of Linguistics, Ghent University, Ghent, Belgium

**Keywords:** V3, V2, cartography, complementizers, Flemish, Ghent

## Abstract

The goal of our paper is to provide a description of an apparent V3 pattern which is salient with some speakers of the Ghent dialect, illustrated in (1), from Vanacker (1980).

1. Vroeger, die bakten wij vier soorten brood
       formerly die baked  we four sorts    bread
       “We used to bake four kinds of bread.” (Gijzenzele 0.28) (Vanacker, 1980, p. 76)

In such examples, what would be an initial adverbial constituent in the root clause *vroeger*, (“formerly”) is separated from the finite verb by what Vanacker (1980) labels a “pleonastic” element, die, in effect leading to a superficial V3 order. At first sight, this element die is optional and it has no impact on the truth conditions of the proposition that it introduces. (2) is also acceptable in the dialect.

(2) Vroeger bakten wij vier soorten brood.
        formerly baked we four sorts    bread
        “We used to bake four kinds of bread.”

In the first part of the paper, we will provide a description of the distribution of die. We will also compare its distribution with that of the more widely distributed resumptive adverbs *dan* (“then”) and *daar* (“there”), which are typical of the Germanic V2 languages (Salvesen, 2016). Our account will be based both on authentic data drawn from corpora and from anecdotal observations as well as on the results of elicitations with 10 native speakers of the dialect. In the second part of the paper we provide an analysis in terms of Wolfe's (2016) typology of the syntax of V2. Adopting the articulated structure of CP as elaborated in the cartographic framework, we will propose that die is an overt spell out of the head Force and as such a root complementiser.

## 1. Aim and scope of the paper

### 1.1. Background: adverbial V3 resumption in V2 languages

It has been noted in the literature that the adverbial resumption pattern in (1), illustrated for a range of Germanic languages, is a striking property of V2 languages. In this pattern, an initial adverbial modifier is followed by a resumptive element and by the finite verb. While linearly a V3 pattern, the availability of this adverbial resumptive pattern seems to correlate with the V2 property. The resumptive pattern does not occur in languages that do not have a V2 structure (Salvesen, 2016, p. 1). The resumptive adverbial element is optional; its absence yields the typical V2 pattern.


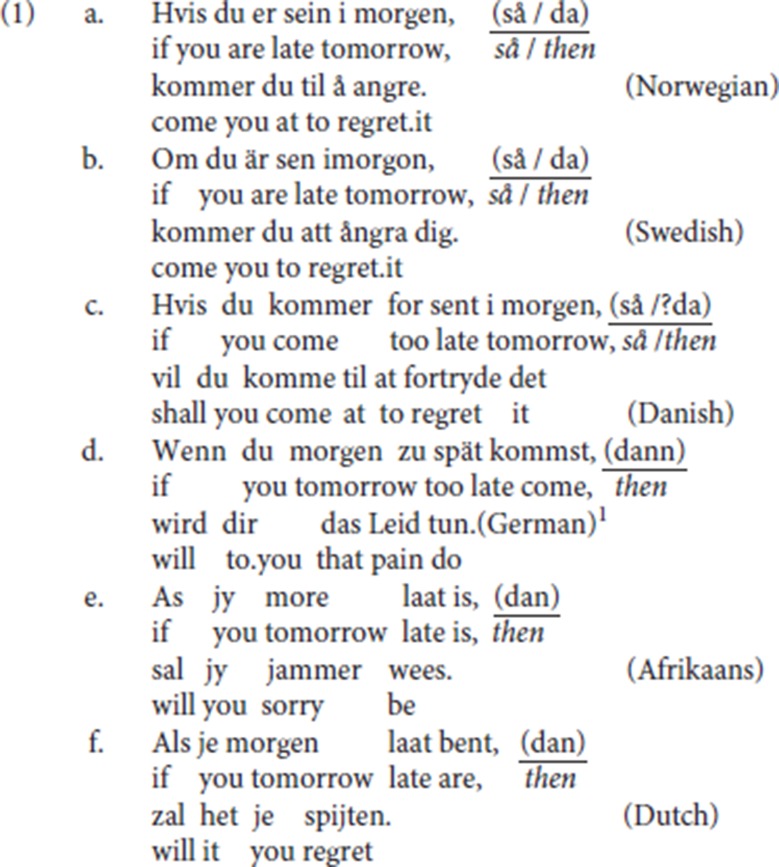


(1) illustrates two types of adverbial resumptive elements, which Salvesen (2016, pp. 4–5) distinguishes as generalized resumptives vs. specialized resumptives. Patterns with a generalized resumptive are illustrated by the resumptive *så* (“so”) in mainland Scandinavian (1a–c): generalized resumptives take the form of adverbial elements that have undergone semantic bleaching, and they may be preceded by a wide range of adjuncts. Languages with a generalized resumptive also have access to specialized resumptives. In patterns with specialized resumptives, the resumptive element is an adverbial element that retains its original meaning. In their resumptive use, these adverbs match the semantics of the initial adjunct. In the mainland Scandinavian data (1a)–(1c), the specialized resumptive is *da* (“then”), a temporal or conditional adverb. As illustrated in Norwegian (2), as a result of the matching condition, an initial temporal or conditional constituent is resumed by specialized *da* (“then”) and cannot, for instance, be resumed by *der* (“there”), the resumptive specialized for locative antecedents:


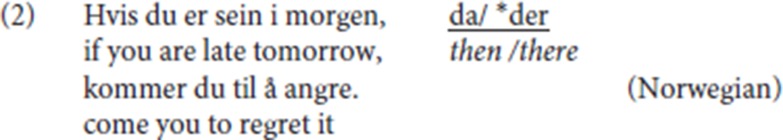


Similar patterns are found in German, Afrikaans and Standard Dutch: in (1d–f) *dann/dan* (“then”) is the resumptive specialized for temporal/conditional antecedents. The matching between the resumptive adverb and the initial constituent is illustrated in Standard Dutch (3): like Norwegian *der* (“there”) in (2), Standard Dutch *daar* (“there”), the locative resumptive, is incompatible with a temporal antecedent. Moreover, Standard Dutch distinguishes between two specialized adverbs, temporal/conditional *dan* (“then”) and temporal *toen* (“then”), which both translate into English as *then*. The adverb *dan* is specialized for future or conditional contexts; the adverb *toen* is specialized for past contexts. This difference is upheld in their specialized resumptive uses: in (3a) the future temporal clause must be resumed by *dan* rather than by *toen*; in (3b) *dan* is inappropriate and the past temporal clause must be resumed by *toen*. See Broekhuis and Corver (2016, p. 1704)^2^ ^3^


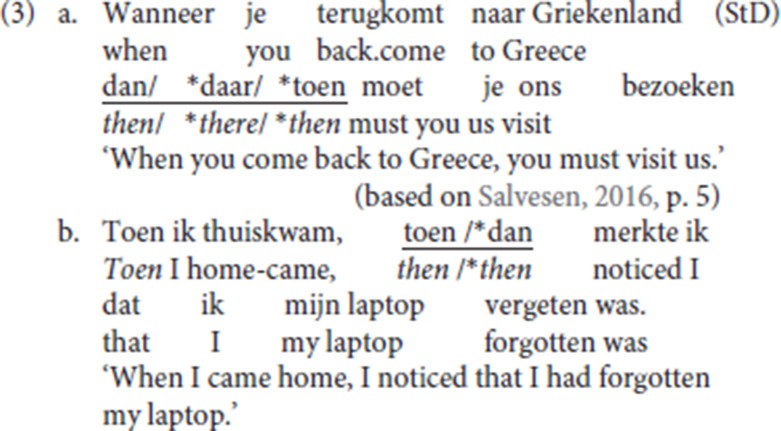


### 1.2. A generalized resumptive in the ghent dialect

The focus of this paper is on the variety of Dutch spoken in Ghent and the surrounding region. The research is based on two transcribed recordings dating from the 1960s (Leemans, 1966; van Hoe, 1981), on anecdotal data collected by the authors, as well as on consultation of native speakers and on elicitation by means of a questionnaire survey of native speakers.

The Ghent variety of Dutch is robustly V2. Nevertheless, a striking property of the dialect and that of the surrounding region is the prolific use of the V3 resumptive pattern illustrated in (4), in which an initial adjunct is separated from the finite verb by an optional connecting particle^4^ *die*, henceforth glossed as die:


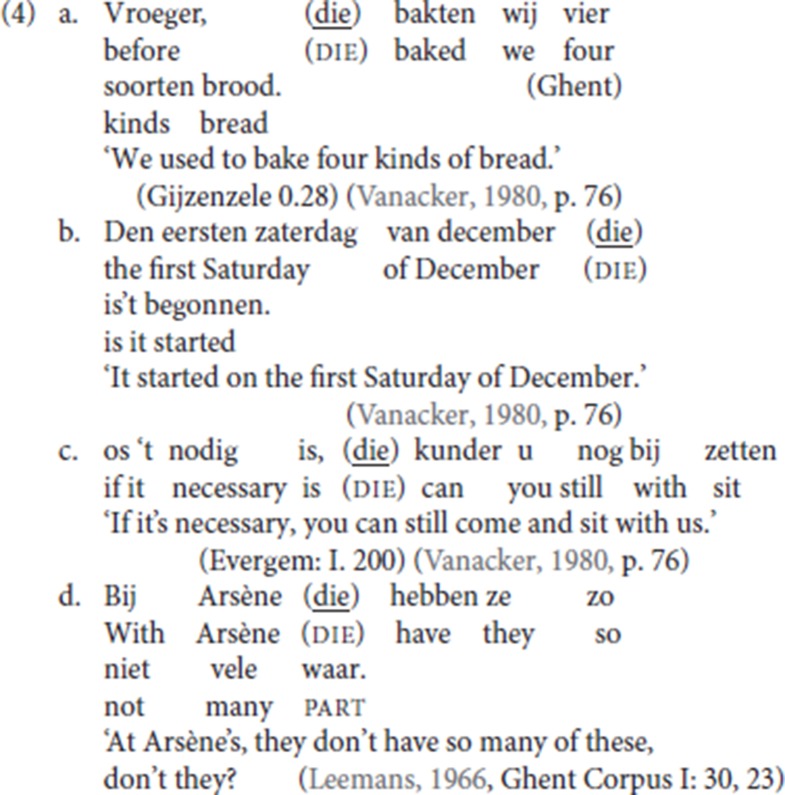


Vanacker (1980) characterizes this particle as a “pleonastic” element^5^. At first sight, the particle die is a semantically bleached element used as a generalized resumptive. As seen in (4), die can follow, among others, a temporal adjunct (4a,b), a conditional adjunct (4c) and a locative adjunct (4d). The particle has no obvious English counterpart. In what follows, this resumptive use of *die* will be referred to as “pleonastic die.”

As already mentioned, pleonastic die is optional: it can always be omitted without loss of grammaticality. Truth-functionally, the omission of pleonastic die has no effect. Pleonastic die is immediately followed by the finite verb, which itself precedes the subject. This entails that the finite verb must have moved to a left-peripheral position. Since in the Ghent dialect movement of the finite verb to the left periphery is a root phenomenon (on root phenomena see Hooper and Thompson, 1973; Emonds, 1976; Haegeman, 2012 a.o.), it follows that pleonastic die is a root phenomenon.

Though the exact geographical spread of the use of pleonastic die remains to be determined, the analogs of (4) are ungrammatical in most Dutch and Flemish dialect areas outside of the Ghent dialect, as shown for Standard Dutch, from now on abbreviated as StD, in (5).


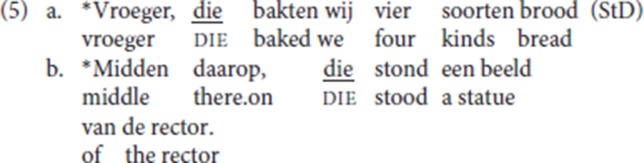


StD and its varieties resort to the specialized resumptive adverbs, cf. (3) and (6) (see Hoekstra, 1999, p. 60; Broekhuis and Corver, 2016, p. 1704):


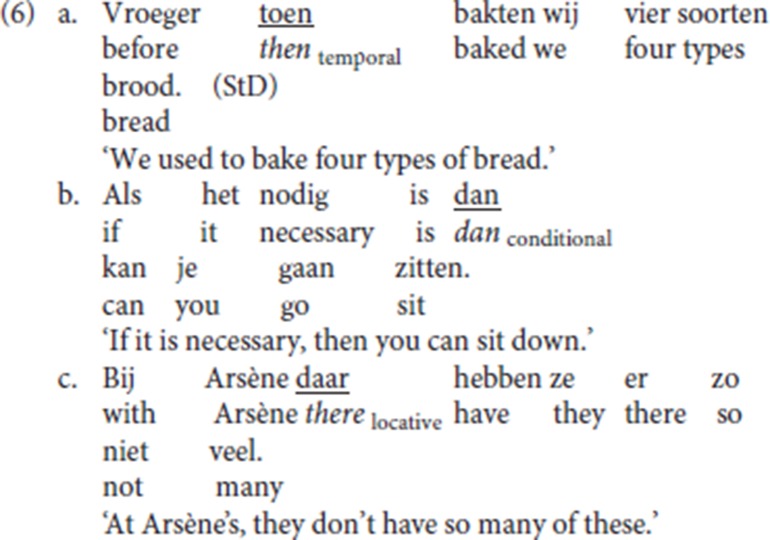


Like other languages with generalized resumptives (cf. Salvesen, 2016), the Ghent dialect also deploys specialized resumptives in addition to the generalized resumptive: in (7a) the temporal adverb *tons* (“then”) is used to resume a conditional adverbial; in (7b), the locative adverb *daar* (“there”) resumes a locative adverbial PP. Both *tons* (“then”) and *daar* (“there”) can also be used as independent adverbs.


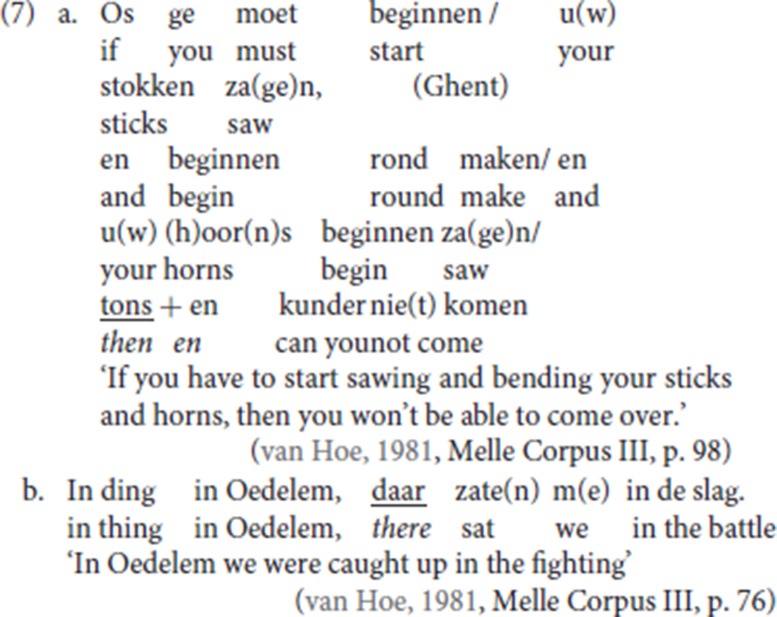


### 1.3. Goal and organization of the paper

Except for a brief discussion in Zwart (1997, pp. 249–250), pleonastic die has so far not been given much attention in the formal literature. This paper will document the pattern and provide an analysis of the data in terms of Poletto's (2013) and Wolfe's (2016) cartographic typology of the syntax of V2. Based on a range of distributional and interpretive properties, we will argue that die is not a phrasal resumptive (as suggested in Zwart, 1997, pp. 249–250) but rather that it has head status and we analyse die as a root complementizer, spelling out a [+declarative] Force head.

In a more general perspective, our paper will reveal that not all resumptive V3 patterns should be assigned the same representation, and in particular that there is micro-variation in relation to the position of the initial constituent in such patterns, which may be main clause-external or main clause-internal, and also in the left-peripheral position of the finite verb. The paper will also show that at least in the Ghent dialect the generalized resumptive has a different syntax from the specialized resumptive. Finally, the paper offers further evidence for micro-variation in the syntax of V2.

The paper is organized as follows: section 2 discusses the properties of the constituent immediately preceding pleonastic die, referred to here as the antecedent. Section 3 briefly inventorizes other pronominal uses of *die* in the dialect, focusing on its use in Contrastive Left Dislocation (CLD), which most closely resembles the pleonastic die pattern. Section 4 contrasts the use of pleonastic die with that of specialized resumptives. Section 5 presents a first cartographic analysis of pleonastic die, proposing that it is a root complementizer merged in the left-peripheral head Force. Sections 6 and 7 explore the predictions of the analysis. Section 7 also refines the analysis and proposes that pleonastic die is a variant form of the declarative complementizer *dat*. Section 8 summarizes the paper.

## 2. The “antecedent” of ghent pleonastic die: an inventory

For convenience, from now on we refer to the constituent immediately preceding pleonastic die as its “antecedent.” The term is used pre-theoretically (cf. section 5).

Vanacker (1980, p. 77) signals the “antecedent requirement” on pleonastic die: the obligatory presence of the antecedent is confirmed both by our corpora and by our informants^6^. In discourse fragment (8), A's utterance provides a potential antecedent for the resumption in B, but as shown by the unacceptability of B's utterance, this is insufficient: die must have an overt antecedent.


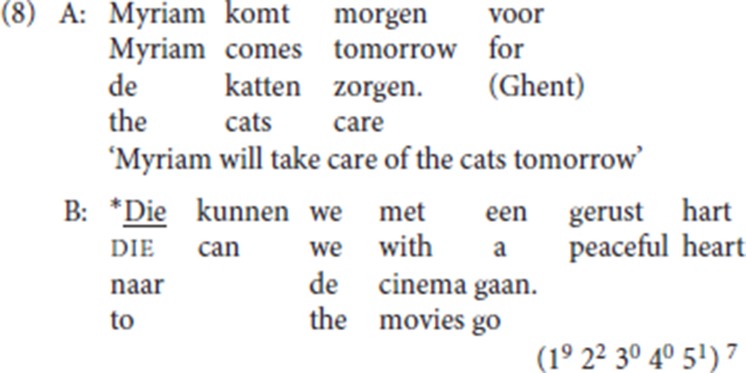


(1^9^ 2^2^ 3^0^ 4^0^ 5^1^) ^7^

In the present section, we inventorize some properties of the antecedent: we will be looking at its syntactic category (section 2.1), its interpretation (section 2.2), its grammatical function (section 2.3) and its distribution (section 2.4).

### 2.1. The syntactic category of the antecedent

As seen in (4), the antecedent of pleonastic die can be realized by different syntactic categories, such as an adverbial phrase (4a), a nominal with adverbial meaning (4b), an adverbial clause (4c), and a PP (4d). In section 6.2.1 we will provide additional evidence that the antecedent of pleonastic die is phrasal.

### 2.2. The interpretation of the antecedent

The adjunct immediately preceding pleonastic die may have a range of (adverbial) interpretations: in (4a) and (4b) the antecedent is temporal, in (4c) it is conditional, in (4d) it is locative. To further illustrate the wide semantic range of the antecedents of pleonastic die, we add the examples in (9). In (9a) the antecedent is a goal adverbial, in (9b) it is a linking adverb, in (9c) it is an expression of evidentiality providing the source of the information, in (9d) it is an epistemic modal adverb.


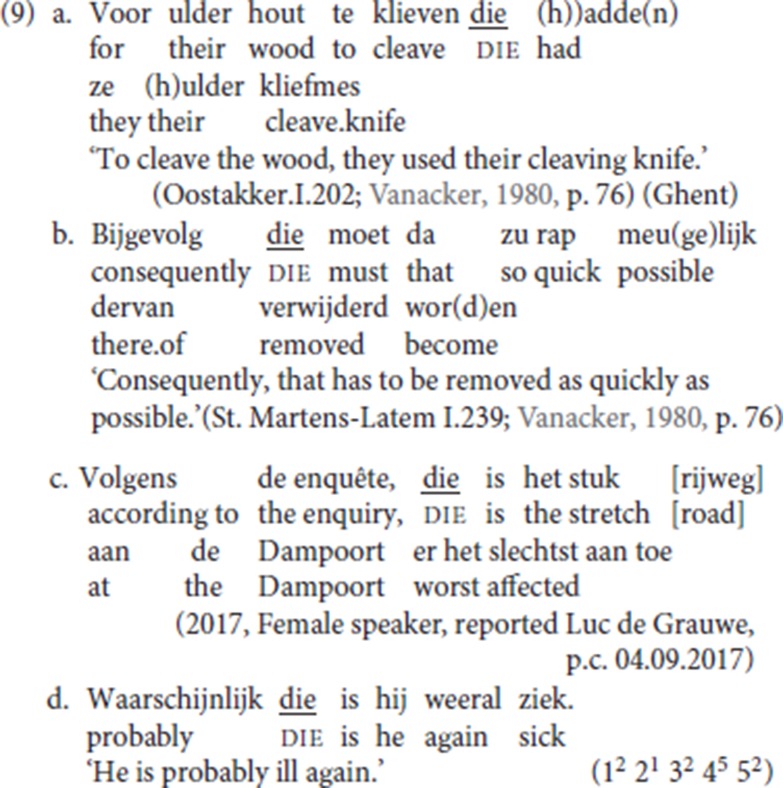


In the StD Contrastive Left Dislocation pattern, the antecedent of the resumptive element is systematically a discourse familiar topic (cf. de Vries, 2009; Den Dikken and Surányi, 2017, p. 547) and an epistemic adverb such as *waarschijnlijk* (“probably”) would be disqualified as an antecedent (cf. Broekhuis and Corver, 2016, p. 1707, on *waarschijnlijk*). This suggests that an analysis of pleonastic die in terms of an adverbial variant of CLD would not be appropriate. The acceptability of (9d) also sheds doubt on Zwart's proposal (1997, pp. 249–250), to which we return in sections 3.2 and 4, according to which die would be the specifier of a left-peripheral topic head.

### 2.3. The grammatical function of the antecedent

#### 2.3.1. Argumental PP

In the corpus, most antecedents to pleonastic die can be characterized as “optional” adjuncts in the sense that they do not realize the thematic roles of the main predicate. However, selected arguments are also resumed by pleonastic die. We provide some relevant data here.

First, the corpus contains examples in which pleonastic die follows a locative argument. The following are relevant examples:


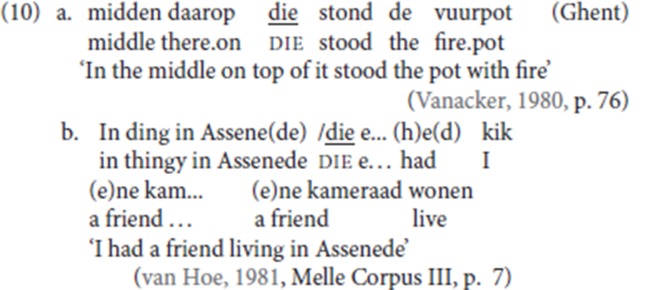


The majority of our informants accept some or all of the examples in (11a), (11b), and (11c) with an argumental PP antecedent.^8^ Our informant CM scored (11d) 7/7.


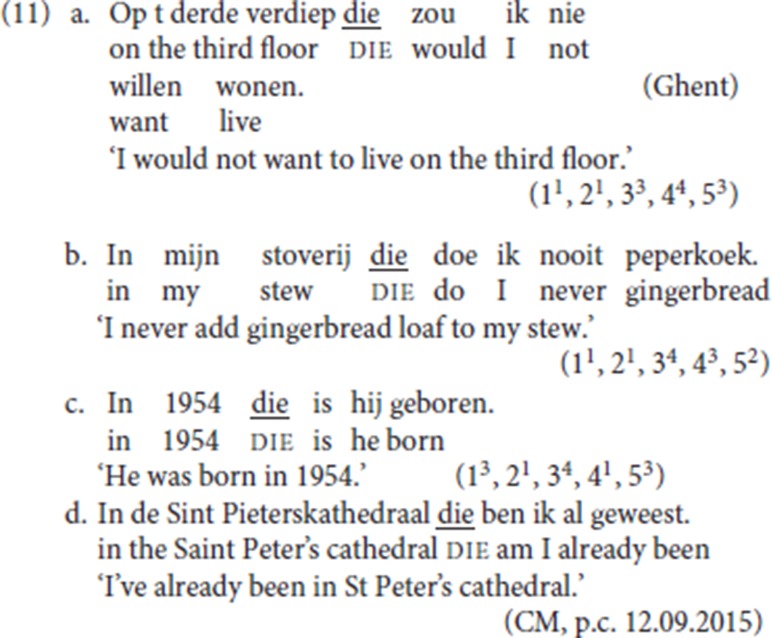


But not only adverbial arguments are available: (12a), from the corpus, illustrates an experiencer PP being reprised by pleonastic die; in (12b) and (12c), both provided by an informant, a PP complement of the verbs, *spreke* (“talk”) and *peize* (“think”) respectively, is followed by pleonastic die.


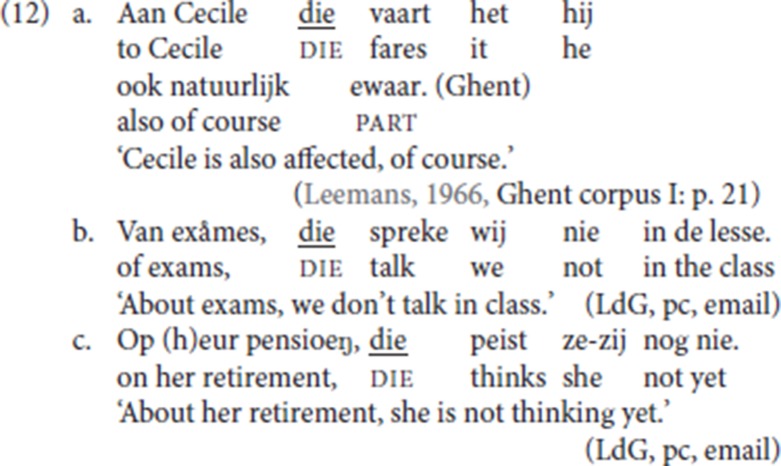


#### 2.3.2. Wh antecedents

For several of our informants, the antecedent of pleonastic die can be a *wh*-constituent: in (13a) the initial constituent *wanneer* (“when”) is a *wh*-adjunct; in (13b) the initial constituent is a nominal *hoeveel* (“how many”).^9^


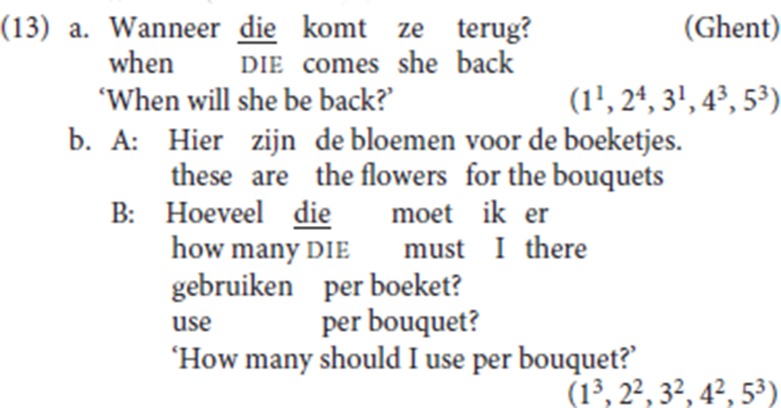


The fact that *wh*-antecedents are potential antecedents for die sheds further doubt on Zwart's (1997, pp. 249–250) analysis which assimilates the pleonastic die pattern to the adverbial variety of left dislocation and according to which die would systematically be the specifier of a left-peripheral topic head: at first sight, it would be difficult to envisage the *wh-*constituent as the antecedent of a topical resumptive. We return to this point in section 4.

### 2.4. The position of the antecedent of *die*

When the antecedent of pleonastic die is a *wh*-phrase (13), the *wh*-phrase contributes to the encoding of illocutionary force, and hence it cannot be main clause-external (in the sense of Broekhuis and Corver, 2016, pp. 1133–1134) or “extra sentential” (Astruc-Aguilera, 2005): typically (see Haegeman and Greco, 2018), main clause-external constituents are added onto a sentence which already has illocutionary force and they cannot themselves encode the illocutionary force of the associated clause. Only if the antecedent of pleonastic die occupies a clause-internal left-peripheral position will it be able to encode illocutionary force. Argumental antecedents (cf. section 2.3.1) can also be taken to originate in a TP-internal thematic position.

(14a) shows that the antecedent of pleonastic die can reconstruct for scope: the initial temporal PP *over drie jaar* (“in three years time”), which appears to the left of pleonastic die, modifies the time of the activity encoded by the lexical verb *verhuizen* (“move”), itself the complement of the modal *willen* (“want”). The non resumptive pattern is given in (14b).^10^


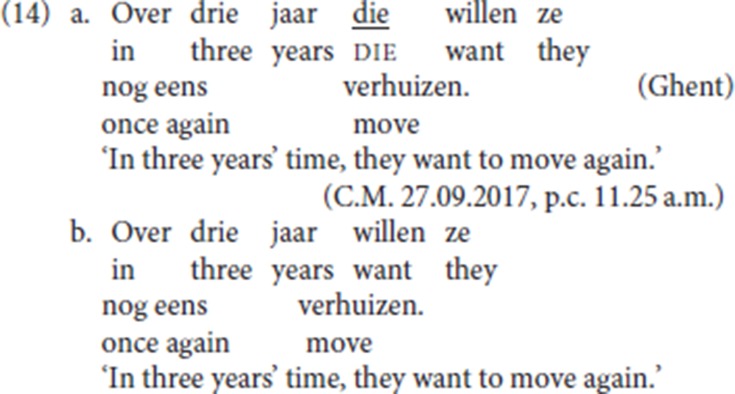


As shown extensively in Haegeman and Greco (2016, 2018), main clause-external adjuncts that give rise to V3 patterns in West Flemish do not reconstruct to lower positions. We refer to Haegeman and Greco (2018) for full discussion of reconstruction patterns.

## 3. Other uses of the formative *die* in the ghent dialect

In the Ghent dialect, the formative *die* has a number of additional (though related) uses. Unlike the specific use of pleonastic die focussed on here, these uses are shared by other varieties of Dutch. In all these uses, *die* could be said to have nominal features: it is involved in the encoding of referential and coreferential relations, being used for instance as a distal demonstrative or as a relativizer. In such uses, *die* is gender-sensitive: it has gender-based inflection and it alternates with *dat*. For reasons of space we cannot discuss these uses of *die* in detail; we will provide a short overview and then focus on the resumptive use in the Contrastive Left Dislocation pattern, which we abbreviate as CLD.

### 3.1. Overview

(15) illustrates some nominal uses of the formative *die*. First, *die* is part of the paradigm of the demonstrative determiner, as shown by *die cafes* (“those pubs”) in (15a) and *dienen tijd* (“that time”) in (15b). As shown by these examples, the demonstrative is inflected for gender, with *die* in (15a) the plural form and *dienen* in (15b) the masculine singular form. Pleonastic die does not manifest gender inflection^11^.

In addition, *den diene* in (15b) illustrates the use of the distal demonstrative as an independently referring expression: in this use, *die* is preceded by an article (i.e., *den diene*). Again, the alternation between masculine singular *den diene* and feminine singular or plural *de die*, illustrated in (15c), *is* gender based. The neuter form is *dat* (but also Rullman and Zwart, 1996 for a more nuanced view on the use of *dat*). As seen in (15c), the “strong form” *de die*, combining the determiner and the demonstrative, alternates with a short form *die*. The latter is invariant for gender and does not alternate with *dat*.

The second occurrence of *die* in (15a) with the form *dien*, illustrates its use as a relative pronoun. This form displays complementizer agreement: the plural ending –*n* matches the plural relativized subject *die cafes* (“those pubs”). Complementizer agreement is also instantiated on the subordinating conjunction *dat* (“that”), as illustrated in (15d).


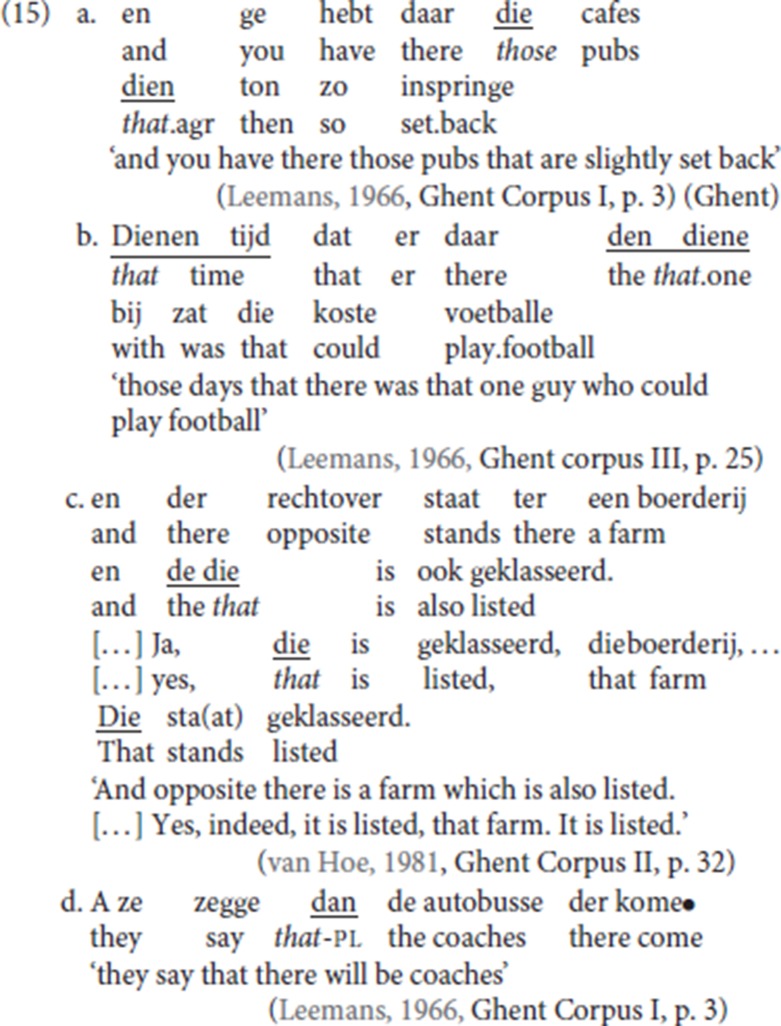


We will not dwell further on these manifestations of the formative *die*. The core points to retain are that pleonastic die does not alternate with *dat*, is not inflected for gender and does not display complementizer agreement^12^.

### 3.2. Contrastive left dislocation

As mentioned, in the Ghent dialect, the formative *die* is also used in Contrastive Left Dislocation (CLD): in this pattern an initial constituent is reprised by a resumptive pronominal belonging to the demonstrative paradigm (cf. Broekhuis and Corver, 2016, pp. 733–734/1328/1457/1691; Den Dikken and Surányi, 2017). In view of our later discussion, we distinguish three types.

#### 3.2.1. CLD with a DP antecedent

(16)a illustrates StD CLD: the dislocated nominal constituent *Jan* is resumed by the demonstrative *die*. Like examples with pleonastic die, CLD instantiates V3 order. As shown in (16b), *die* alternates with *dat*, the alternation being, among other things determined by gender, *dat* being neuter, and by semantic properties (see Rullman and Zwart, 1996).


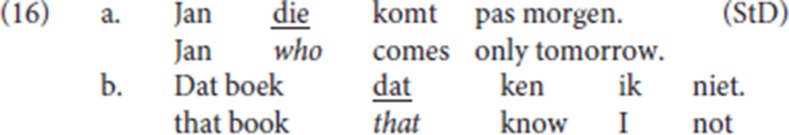


For CLD in the Ghent variety, two types of resumption are found in our corpus, reflecting the two forms of demonstrative *die* as a referential demonstrative as illustrated in (15b) and (15c) in section 3.1. In the first pattern, (17a), the CLD resumptive is the “strong” variant of the demonstrative which combines determiner and demonstrative, with *dat* the neuter alternative (not illustrated, see note 14). The majority of CLD cases in the corpus illustrate the second pattern (17b), with the “short” form of the demonstrative *die*. For the second pattern, there is no gender-matching: even with a neuter antecedent, the form *die*, rather than the form *dat*, is used, as illustrated in (17c). In this respect, the Ghent dialect differs from most other Flemish dialects, in which gender-matching is maintained^13^.


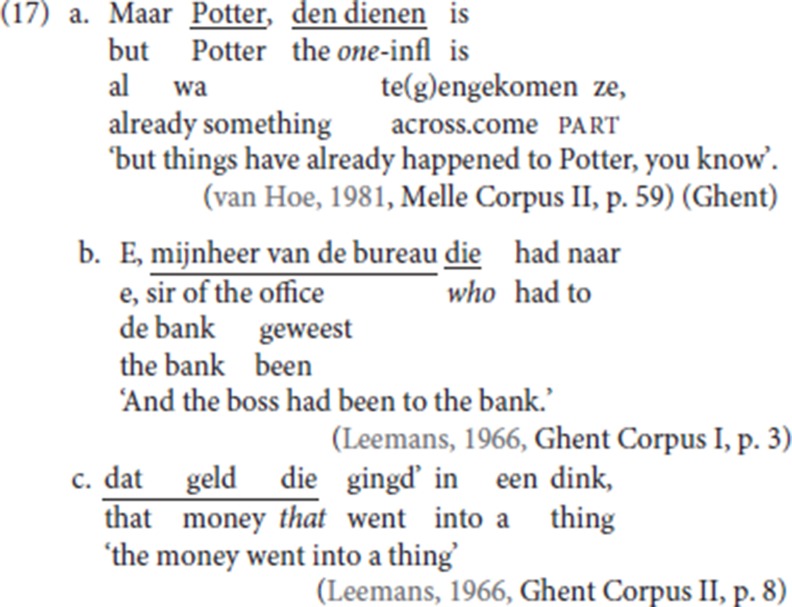


Resumptive *die* can also pick up a bare quantified nominal (18a, b), which has been reported as unacceptable for Dutch CLD^14^.


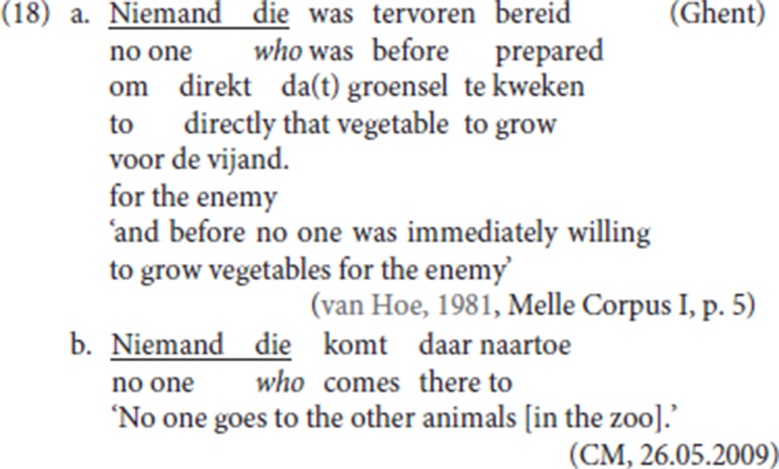


#### 3.2.2. CLD, PP antecedents and P-stranding

In StD (19), an initial PP (*over examens* “about exams,” *aan haar pensioen* “about her pension”) is resumed by the R-word *daar* (“there”), itself the complement of the stranded preposition. In line with the literature on Dutch (see a.o., van Riemsdijk, 1978; Koopman, 2000, 2010; Noonan, 2017), we assume that P-stranding is derived by movement of the resumptive R-pronoun *daar* (“there”) from the complement position of the preposition.


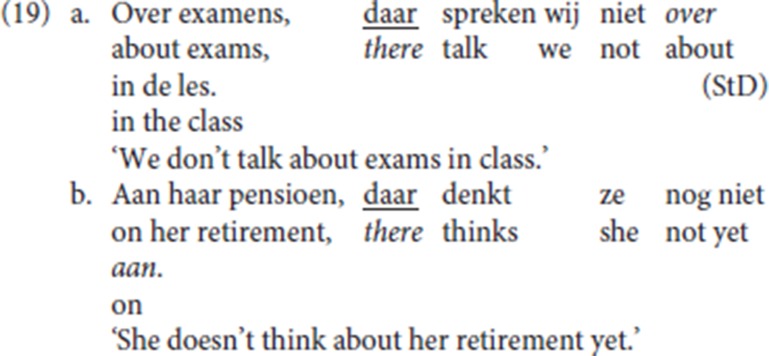


(20) is the Ghent analog of (19) (LdG, p.c. email): the fronted resumptive *daar* strands the associated preposition [*van* (“of”) and *op* (“on”)], and it is anteceded by a PP or by a DP. Anticipating the discussion in section 4.6 below [see the data in (32)], with P-stranding pleonastic die is not available.


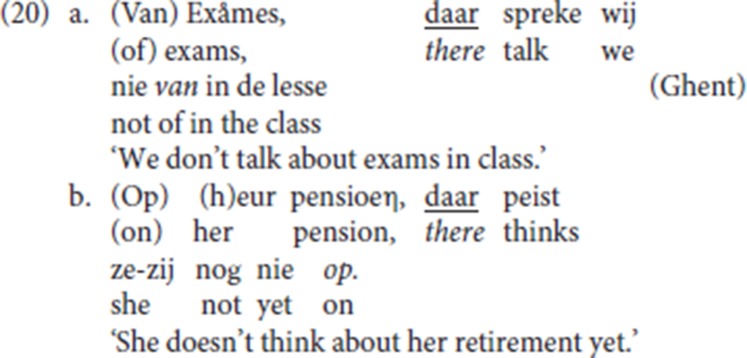


#### 3.2.3. CLD with an adverbial antecedent

It seems reasonable to follow Hoekstra (1999, p. 60) and Broekhuis and Corver (2016, p. 1704) and analyse StD and Ghent V3 patterns in which an adverbial adjunct is picked up by a specialized resumptive adverb as the “adverbial” variant of CLD: StD (3a,b) are repeated as (21a,b), (6c) from the Ghent dialect is repeated as (21c)^15^.


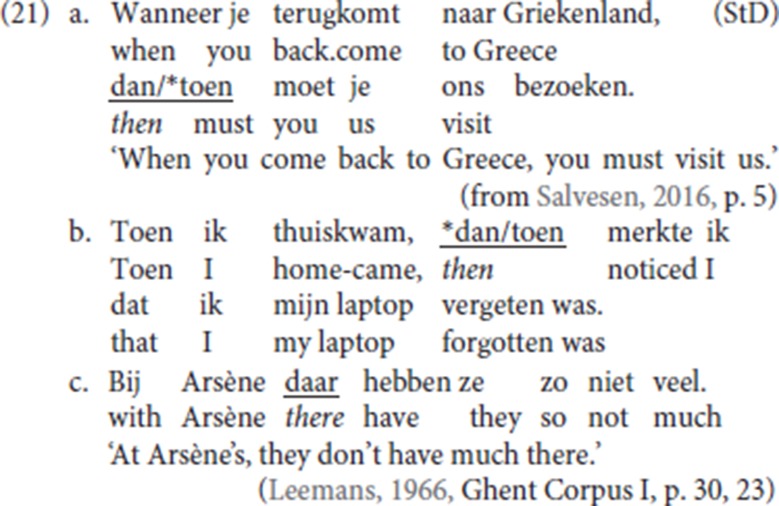


## 4. Pleonastic die vs. the specialized resumptive in adverbial CLD

This section compares resumption with pleonastic die with the CLD pattern, focusing on the CLD pattern with the specialized adverbial resumptive illustrated in section 3.2.3 and on the CLD pattern with P-stranding illustrated in section 3.2.2.

We assume—in line with Hoekstra (1999, p. 60) and Broekhuis and Corver (2016, p. 1704)—that StD adverbial resumption is a variant of CLD with an initial adjunct and a fronted specialized resumptive in the sense of Salvesen (2016). We assume that this analysis carries over to resumption with the specialized adverbs [*dan* (“then”), *daar* (“there”) etc.] in the Ghent dialect. Zwart (1997, pp. 249–250) assimilates the Ghent pleonastic die pattern with pronominal *die* in left dislocation and proposes that the pleonastic die element is the specifier of a left-peripheral topic projection. Pursuing this line of reasoning, it would then be tempting to also unify the syntax of pleonastic die with that of adverbial CLD with a specialized resumptive. However, in what follows we will show that assimilating the two patterns fails to capture the contrasts between specialized resumptive adverbs and pleonastic die in the Ghent dialect; we will analyse the specialized resumptive adverbs as phrasal constituents in a left-peripheral specifier position (in line with Zwart's proposal for pleonastic die), but we will analyse pleonastic die as a left-peripheral head.

### 4.1. Distribution

In both StD and in the Ghent dialect, specialized resumptive adverbials like temporal *dan* (“then”) can appear in a middle field position: this pattern arises whenever the dedicated left-peripheral slot is unavailable because an additional left-peripheral feature is independently activated; the relevant pattern is illustrated in (22). (22a) is the default pattern in which the initial conditional clause is resumed by the specialized adverbial resumptive *dan*, which occupies the initial position of the V2 clause. Being occupied by a *wh*-phrase, *wat* (“what”) in (22b, c), the initial position can no longer host the resumptive adverb *dan*: therefore, the resumptive adverb cannot precede the finite verb. Instead, the resumptive adverb appears TP-internally (22d). The pattern is replicated with nominal antecedents in CLD, see for instance Den Dikken and Surányi (2017, p. 551, (14c)). In the Ghent dialect too, specialized adverbial resumptives occupy a mid position (22e, 22f) whenever the left-peripheral slot is unavailable.


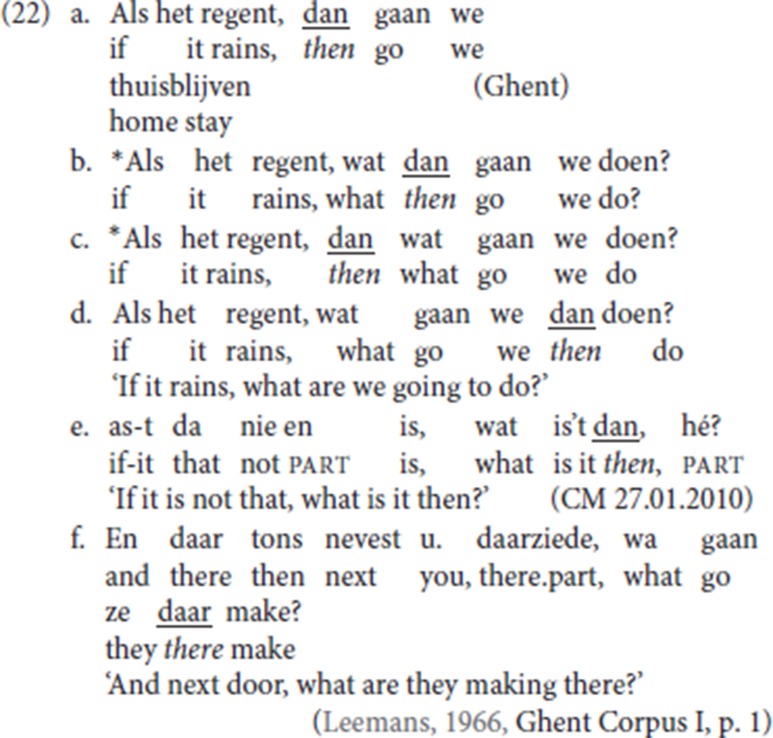


From (22) we infer that fronted specialized resumptive adverbs are in complementary distribution with fronted *wh-*operators. We assume that the resumptive adverbs are operators which by default target a left-peripheral position. This entails that, like their non resumptive counterparts, specialized resumptive adverbs are phrasal and that their first merge position is TP-internal. We thus adopt a movement analysis for the derivation of adverbial CLD. If the fronted specialized resumptive adverbs target the same left-peripheral position as fronted *wh*-phrases, it will follow that they do not themselves take *wh*-phrases as their antecedent. For some discussion of the interaction between such operators and the left periphery of the clause see also Mikkelsen (2015) and Haegeman and Greco (2018).

For pleonastic die, on the other hand, a TP-internal position is unavailable^16^. In (23a) and in (23b), the left-peripheral initial position hosts the *wh-*constituent [*wat* (“what”), *hoeveel* (“how many”)] but nevertheless die cannot occupy a lower position. This piece of evidence already suggests that the syntax of pleonastic die, a generalized resumptive, cannot be fully assimilated to that of its specialized counterparts. In both cases replacing die by *dan* leads to fully acceptable examples (23c,d).


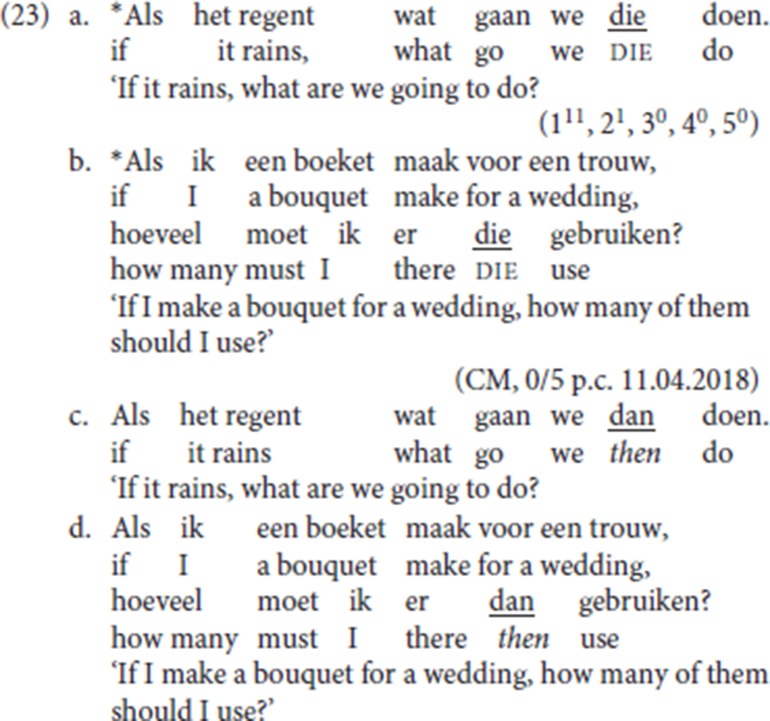


The evidence in (23a) and (23b) is not as clear cut as one would wish:^17^ both (23e) and (23f) with die to the immediate right of the *wh*-phrase are also judged as degraded:


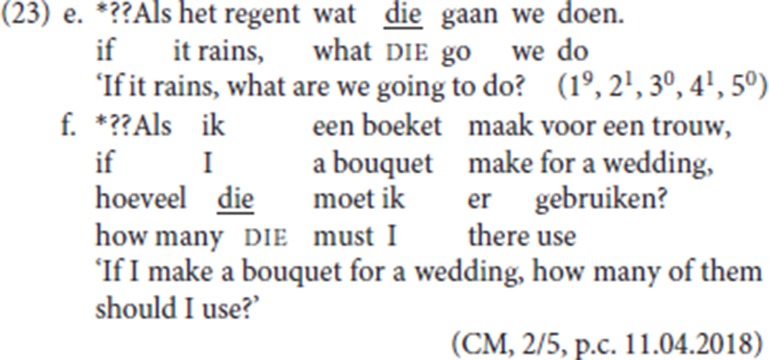


Interestingly, (23f) contrasts with (13b), which also features pleonastic die to the right of the *wh*-phrase *hoeveel* (“how many”) and which was scored as follows: 1^3^, 2^2^, 3^2^, 4^2^, 5^3^, i.e., with 7 speakers rating the sentences as (relatively) acceptable. We suspect that the added complication which leads to the degradation in (23e) and (23f) is that these examples feature an initial conditional clause to the left of a fronted *wh*-phrase. Anticipating the analysis elaborated in section 5, we assume that the initial adverbial constituent in (23e) and (23f) occupies a main clause-external position. Our tentative hypothesis is that because of its main clause-external position combined with the presence of the *wh-*constituent, the initial conditional clause cannot be interpreted as a modifying the main clause modality. For discussion of the interpretation of initial adjuncts in relation to V2 clauses see Haegeman and Greco (2018).

In section 4.6 we offer additional evidence from P-stranding that pleonastic die is incompatible with a TP-internal position. The incompatibility of pleonastic die with the TP-internal position might be due to the fact that while pleonastic die is merged TP-internally, some specific discourse-related feature forces it to move to the left periphery. The relevant feature could be similar to, say, a *wh*-feature or the operator feature on relative pronouns. Alternatively, the fact that a TP-internal position is unavailable could be due to the fact that pleonastic die is not merged TP-internally at all but is merged directly in the left periphery. Below we will pursue the latter option (see section 4.6).

### 4.2. Antecedent requirement

Adverbs deployed as specialized resumptives such as *dan* (“then”), *toen* (“then”) or *daar* (“there”) can be used independently as temporal/conditional/locative modifiers, both in initial position or in TP-internal position^18^. (24) is StD.


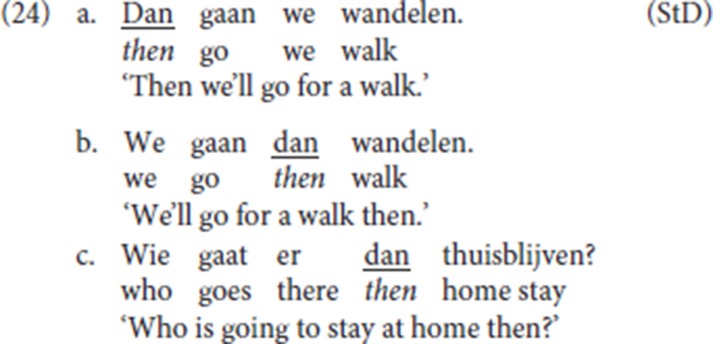


In contrast, as mentioned, pleonastic die cannot be used independently with an adverbial function: it requires an antecedent. This was illustrated in (8), where we showed that even if the context makes an implicit antecedent available, this is insufficient to license the use of pleonastic die.

### 4.3. Type of antecedent

Recall that StD fronted specialized resumptives are not compatible with a *wh*-operator as an antecedent^19^. This follows if fronted specialized resumptive adverbs are left-peripheral operators and target the same operator position as *wh-*operators.


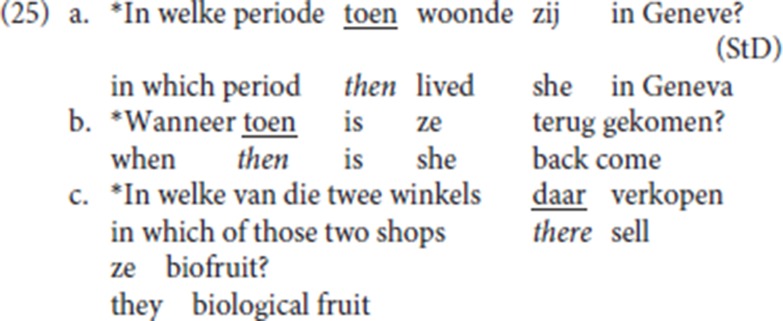


As already shown in the Ghent example (13a), repeated as (26), pleonastic die is compatible with an initial *wh*-adjunct as its antecedent.


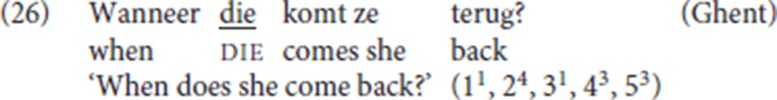


So, while fronted specialized resumptive adverbs compete with a *wh-*operator, pleonastic die does not compete with a *wh-*operator. We take this as a second strong indication of the difference between the specialized resumptive adverbs and pleonastic die.

Recall that in addition to *wh-*antecedents, other antecedents such as the epistemic modal adverb *waarschijnlijk* “probably” (9d), a licit initial constituent in the V2 pattern, are compatible with pleonastic die but are incompatible with the adverbial CLD pattern. See Broekhuis and Corver (2016, p. 1707 on *waarschijnlijk*).

### 4.4. Modifiers

The phrasal status of specialized resumptive adverbs is confirmed by the fact that they can be modified by focus particles such as *zelfs* (“even”) or *just* (“exactly, precisely”), as seen in (27/8a). In contrast, pleonastic die cannot be so modified, as seen in (27/28b)^20^.


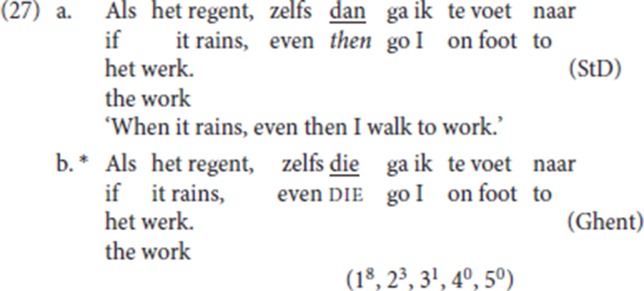



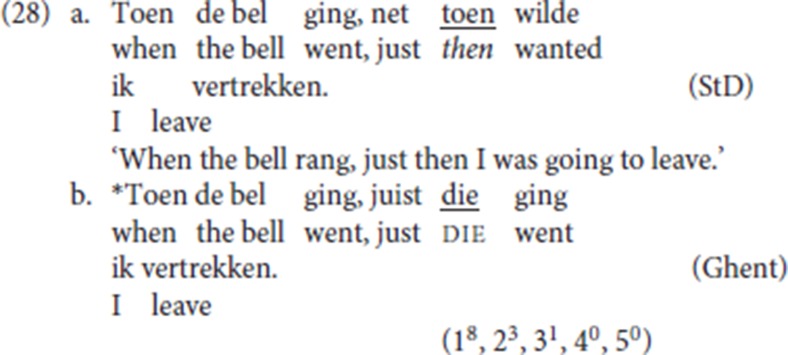


### 4.5. Co-occurrence with specialized (resumptive) adverb

A final confirmation that pleonastic die differs syntactically from the fronted specialized adverbial resumptives and that indeed it occupies a different position comes from the fact that both when used independently (29) or when used as resumptives (30), the specialized adverbs (*dan/toens* “then,” *daar* “there”) can co-occur with pleonastic die: in this case, the specialized resumptive precedes pleonastic die, the alternative order is not available.


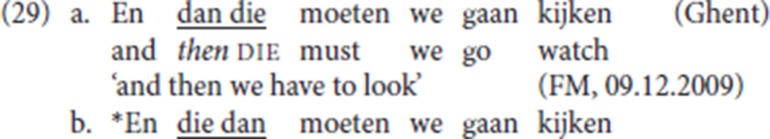



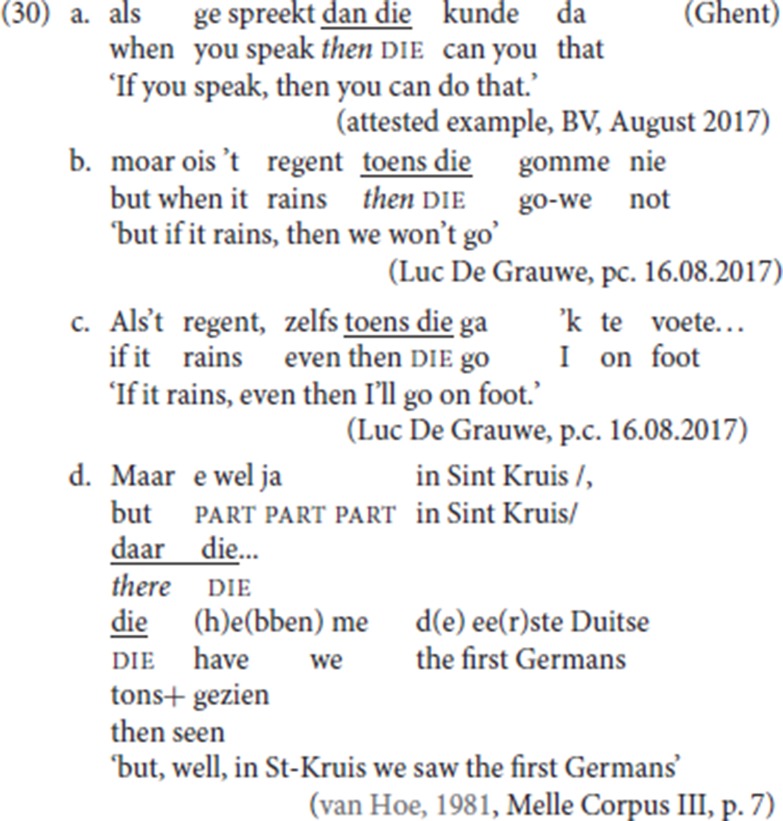


Observe that if the specialized resumptive adverbs in (30) have moved from a TP-internal slot to the left periphery, this suggests that pleonastic die itself has not moved: one would expect that movement of two phrasal constituents would lead to intervention effects. More conclusive evidence against a movement analysis of die will be discussed in the next section.

### 4.6. P-stranding

Recall the StD CLD example (19) and its Ghent analog in (20), which is repeated here as (31), in which an initial PP (*over examens* “about exams,” *aan haar pensioen* “about her pension”) is resumed by the R-word *daar* (“there”), itself the complement of the stranded preposition. We adopt the hypothesis that these CLD patterns are derived by movement of the resumptive element (here the R-pronoun *daar*) from the complement position of the preposition (see Den Dikken and Surányi, 2017 for discussion and evaluation of alternative analyses of CLD).

In the Ghent examples with P-stranding by resumptive *daar* (“there”) (31), the initial constituent can be either a PP (*van exames* “about exams,” *op heur pensioen* “about her pension”), or just a DP [*exames* (“exams”), *heur pensioen* (“her retirement”)], the former a case of CLD, the latter plausibly an instantiation of hanging topic left dislocation (see Cinque, 1977, 1990; Broekhuis and Corver, 2016, pp. 1500–1502 for the difference). For both patterns, our informant LdG signals a prosodic break between the initial constituent and the sentence introduced by *daar*.


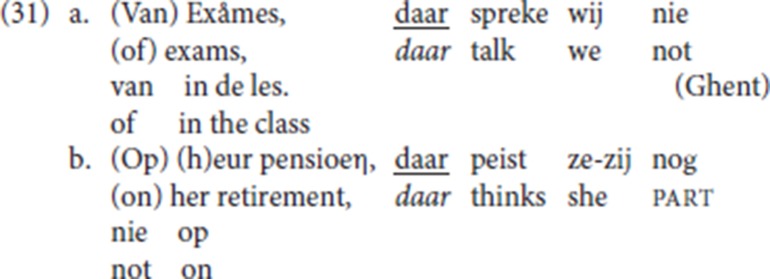


We have shown that pleonastic die also functions in a resumptive pattern in which the initial constituent is a prepositional argument of the verb: (12b-c) are repeated in (32). This pattern differs from that in (31) in at least three ways: (i) no prosodic break is signaled by our informant, (ii) P-stranding itself is not available, and (iii) the initial constituent cannot be the nominal, it must be a PP. While the P-stranding facts in CLD (31) make it plausible that *daar* (“there”) is an operator moved from the complement position of the preposition, this analysis is thus not plausible for pleonastic die in (32).


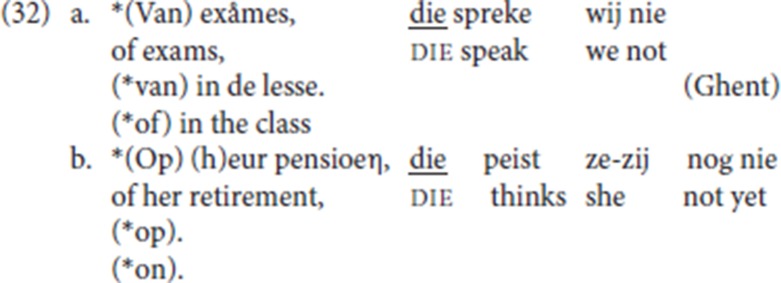


We formulate the hypothesis that pleonastic die is a head, merged directly in the left periphery. Recall that on the basis of reconstruction effects, we also proposed in section 2.4 that the antecedent of pleonastic die is moved to the left periphery from a TP-internal position^21^.

### 4.7. New information focus

The availability of pleonastic die with *wh*-antecedents (cf. sections 2.3.2 and 4.3 and footnote 10) challenges the analysis of its antecedent as a topical element. This is confirmed by the fact that our informants also accept pleonastic die with an antecedent that provides the answer to a *wh*-question.


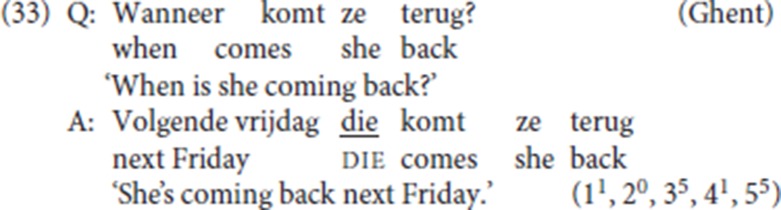


(33) sheds further doubt on Zwart's (1997, pp. 249–250) proposal that pleonastic die is the specifier of a topic head in the left periphery. If anything, in (33) the initial constituent in the answer should be associated with a focus value.

### 4.8. Summary

Table 1 summarizes the contrasts discussed in the preceding section between the specialized resumptives such as StD *dan* “then” and the Ghent variant *tons* “then” on the one hand and pleonastic die; it also correlates the contrasts with the following analytical hypotheses:

- Specialized resumptives
  - specialized resumptives are phrasal constituents, more specifically they are operators;
  - specialized resumptives are moved from a TP-internal position to a left-peripheral operator position (and hence compete with fronted *wh*-constituents).
- Pleonastic die
  - pleonastic die is a head;
  - pleonastic die is merged in a left-peripheral position.

**Table 1:** Specialized resumptive (*dan/tons/demonstrative pronoun*) vs. generalized die.

	**Specialized resumptive**	**Generalized die**
**PATTERNS**		
Middle field position (*wh*/imperative)	Yes	No
Antecedent requirement	No	Yes
*wh* antecedent	No	Yes
Focal modifiers	Yes	No
P stranding	Yes	No
**OUR HYPOTHESES**		
Categorial status	Phrasal (topic) operator	Head
Derivation of Left-peripheral position	Internally merged	Externally merged

We elaborate our head analysis of pleonastic die in sections 5–7.

## 5. A first cartographic analysis of pleonastic die

This section outlines our analysis of the pleonastic die pattern in the Ghent dialect. Because of the differences diagnosed between pleonastic die and the specialized adverbial resumptives, we will not fully assimilate the syntax of pleonastic die to that of an adverbial CLD pattern.

- A fronted specialized resumptive adverb can co-occur with pleonastic die. This entails *de facto* that what would be a generalized resumptive, i.c. pleonastic die, cannot be taken to occupy the same position as the fronted specialized resumptive adverb.
- The constituent to the immediate left of pleonastic die, its ‘antecedent’, can be a *wh*-phrase: this entails that the antecedent cannot be main clause-external.
- Pleonastic die is incompatible with a TP-internal position and with P-stranding: this leads us to the hypothesis that it is not first merged TP-internally and moved to the left periphery, but rather that it is first merged as a left-peripheral head.

### 5.1. Theoretical background: the typology of V2 languages

If the antecedent of pleonastic die is merged in a clause-internal position and moves to the left periphery, we need to postulate at least three positions in the clausal left periphery to derive the V3 pattern:

- a phrasal position for the antecedent phrase;
- a head position for pleonastic die;
- the landing site for the finite verb (which precedes the canonical subject position): a head position.

To accommodate these positions, we explore cartographic proposals for an enriched CP structure (Rizzi, 1997). We will not go into the details of or motivation for the cartographic framework; the present section simply outlines the assumptions that our analysis of the pleonastic die pattern will be based on.

In line with the cartographic elaboration of the left periphery (Rizzi, 1997), the CP structure is decomposed: the lowest projection in the CP layer is FinP, a projection whose head encodes the finiteness properties of the clause, and the projection which closes off the sentence is ForceP, the projection encoding illocutionary force.

There are several cartographic implementations for the analysis of V2 (see Haegeman, 1996; Poletto, 2013; Biberauer and Roberts, 2014 and many others). For our current discussion we will adopt proposals by Poletto (2013) and specifically the implementation in Wolfe (2015, 2016) for the typology of V2 languages. According to these authors, V2 languages are diversified according to the left-peripheral locus targeted by the finite verb, which is either Fin or Force (Poletto, 2013; Wolfe, 2015, 2016 for motivation). Thus, a distinction is made between so called Fin-V2 languages, with the left-peripheral structure schematized in (34a) and Force-V2 languages, whose left periphery is schematically represented in (34b).


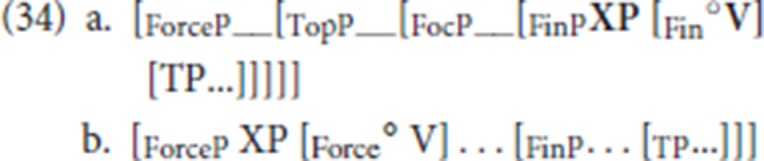


One of the predictions of the Poletto/Wolfe typology is that in Fin-V2 languages, multiple access to the left periphery remains potentially available, leading to the attestations of V3 and V4 orders. This prediction is explored for medieval Romance in Benincà and Poletto (2004) and Benincà (2004, 2006, 2013). On the other hand, in Force-V2 languages, a V3 pattern only arises when what would in effect be main clause-external constituents (in the sense of Broekhuis and Corver, 2016, pp. 1133–1134) are combined with a full fledged V2 clause, i.e., ForceP. We assume with Haegeman and Greco (2018) that such main clause-external constituents are inserted in a functional domain outside ForceP. According to Haegeman and Greco (2016, 2018), the West Flemish and StD V3 patterns involve constituents merged in a main clause-external projection, labeled FrameP, as schematized in (35).


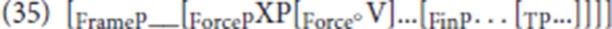


In our paper we adopt Haegeman and Greco (2018) assumptions about the nature of FrameP. Specifically, FrameP, their discourse structuring projection, corresponds to a number of similar proposals in the literature, including, among others, Emonds's (2004) DiscourseP, Cinque's (2008, pp. 118–119) HP (also adopted in Giorgi, 2014; Frascarelli, 2016), Koster's (2000) :P, de Vries's (2009) and Griffiths and de Vries's (2013) ParP. Following Haegeman and Greco (2018), our representation of the V3 pattern in (35) thus departs from that of authors who analyse a V3 configuration as a further extension of the “Rizzian” left periphery (cf. Holmberg, 2015) and is more in line with Cinque's conception of his HP:

In the spirit of Williams (1977), we must also assume that the ‘Discourse Grammar’ head H, as is the general rule for sentences in a discourse, blocks every ‘Sentence Grammar’ relation between its specifier and complement (internal Merge, Agree, Binding, etc.), despite the asymmetric c-command relation existing between the two under the extension of the LCA to Discourse Grammar (Cinque, 2008, p. 119).

This is not the place to develop the analysis further, but we refer the reader to Haegeman and Greco (2018) for extensive discussion and motivation^22^.

Overall the word order pattern in the Ghent dialect is like that in StD and in other Flemish varieties of Dutch, with the characteristic inversion pattern where fronting of a non-subject constituent gives rise to subject-finite verb inversion. On the hypothesis that, like StD and like the Flemish varieties of Dutch, the Ghent dialect is a Force-V2 language, regular root V2 sentences in the Ghent dialect are derived by V movement to Force, via Fin, and by movement of a constituent to SpecForce via SpecFin as represented partially in (36). The second position restriction in the V2 pattern is due to a bottleneck effect (Haegeman, 1996; Roberts, 2004; Biberauer and Roberts, 2014; Holmberg, 2015): filling SpecFinP blocks additional left-peripheral movement from within TP^23^. In particular the idea is that because the initial constituent that ends up in SpecForceP moves through SpecFinP this in effect prevents other constituents from also moving to the left periphery as the filler of SpecFinP gives rise to intervention effects. Note that it is also not possible to merge a constituent in a left-peripheral slot above FinP and lower than ForceP since such a constituent will itself block the movement of the constituent from SpecFinP to SpecForceP. If the externally merged constituent is by hypothesis inserted to satisfy a left-peripheral criterial feature, then it will itself not be able to move to SpecForce^24^.


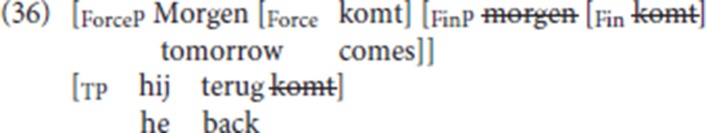


Anticipating the analysis in section 5.2, the pleonastic die pattern in the Ghent dialect will be argued to diverge from other Force-V2 languages: we will propose that in these patterns the finite verb remains in Fin and that Force is realized by die.

### 5.2. The head analysis: pleonastic die as a root complementizer

Based on the differences in distribution between the fronted specialized resumptives in the CLD patterns in the Ghent dialect and pleonastic die, we have concluded that while the former are TP- internal phrasal operators which are moved to the left periphery, pleonastic die is a head first merged in the left periphery. Schematically, we propose that the derivation of the adverbial variety of CLD with a specialized resumptive is as in (37a) and that of resumption with pleonastic die is as in (37b).


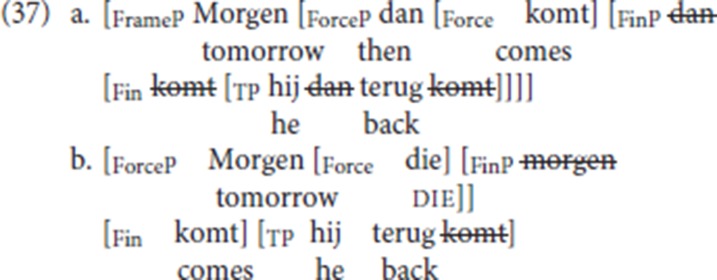


Derivation (37a) can be summarized as follows:

- Following Wolfe (2015), we assume that in Force-V2 languages the left periphery of the V2 clause instantiates two head positions, Force and Fin.
- Fin, the head hosting the finite verb, encodes finiteness properties of the clause. Illocutionary force is encoded on the head Force.
- The finite verb moves via Fin to Force.
- The finite verb spells out the agreement features of Fin, i.e., complementizer agreement.
- The specialized resumptive *dan* corresponds to the initial constituent of the V2 clause: it moves through SpecFinP (cf. Haegeman, 1996) to SpecForceP, thus leading to the bottleneck effect.
- The antecedent of the specialized resumptive, here *morgen* (“tomorrow”) is first merged in a main clause-external projection, which, following Haegeman and Greco (2018) we label FrameP.
- Like the regular V2 pattern in the Ghent dialect, resumption with a specialized adverb is a root phenomenon.

Derivation (37b) can be summarized as follows:

- The left periphery of a pleonastic die sentence instantiates two head positions: Force and Fin.
- Fin, the head hosting the finite verb, encodes finiteness properties of the clause. Illocutionary force is encoded on the head Force.
- The finite verb halts at Fin (to the immediate left of the canonical subject position), and Force is occupied by pleonastic die.
- The finite verb spells out the agreement features of Fin, i.e., complementizer agreement, which therefore is not instantiated on pleonastic die. This accounts for the difference between pleonastic die and relative *die* (cf. section 3.1).
- The initial constituent of the V2 clause, i.e., the “antecedent” of die, moves through SpecFinP (cf. Haegeman, 1996) to SpecForceP, thus leading to the bottleneck effect.
- Like the regular V2 pattern in the Ghent dialect, pleonastic die is a root phenomenon.

For the reader's convenience, we briefly recall that a head analysis of pleonastic die (37b) is based on the following considerations:

- pleonastic die is restricted to the left-peripheral slot: mid position is ungrammatical (section 4.1);
- pleonastic die cannot be modified by focusing particles (section 4.4);
- fronted specialized adverbial resumptives can co-occur to the immediate left of pleonastic die (section 4.5).
- preposition stranding is unavailable with pleonastic die (section 4.6);

Observe that our analysis implies that there is micro-variation in the Force-V2 vs. Fin-V2 typology. In particular, while for the unmarked V2 pattern with the finite verb in linearly second position we assume that the Ghent dialect is a Force-V2 language, in the pleonastic die patterns, the Ghent dialect also manifests a reflex of the Fin-V2 pattern in the sense that the finite verb halts in Fin. In terms of the Fin/Force typology the die patterns are a hybrid in that the finite verb lands in Fin and the spell out of Force is guaranteed by the insertion of die^25^.

We analyze pleonastic die as a filler for a C/Force head in the context in which the finite verb lands in Fin. Given that V movement to Fin is restricted to root environments, this entails that die fills a root C position and hence is a kind of root complementizer (for uses of the complementizer *that* in root clauses in English see Radford, 2018, pp. 156–169). In section 6, we will explore some consequences of the head analysis of pleonastic die. In section 7, we consider some problems and we refine our analysis. One issue we will address in section 7.2 is the question why the “root complementizer” is realized as die rather than as *dat*.

## 6. Exploring the head analysis of pleonastic die

The gist of our analysis is that pleonastic die is inserted in Force and that the obligatory presence of its antecedent is independent of the presence of die itself, but rather results from the Force-V2 requirement. The proposal successfully captures several aspects of the distribution of pleonastic die in relation to other left-peripheral constituents, and, anticipating section 7, it will also capture some initially surprising restrictions.

According to our analysis, the obligatory presence of a constituent to the immediate left of pleonastic die simply follows from the “V2 requirement” on Force. Basically, the Force-V2 requirement translates here as “Force die 2 requirement.” As seen in section 2, pleonastic die does not appear to be selective in terms of the formal or semantic properties of the constituent to its left. Moreover, it is compatible with topical constituents as well as with foci (cf. *wh-*constituents) and with epistemic adverbials.

The analysis has two terminological (and conceptual) implications. One implication of our Force die 2 analysis is that the label “antecedent” is not appropriate for the constituent immediately preceding pleonastic die, because in our conception of the structure, pleonastic die does not “reduplicate” the initial constituent. Rather, the constituent preceding die satisfies Wolfe's (2015, 2016) Force-V2 requirement, the head Force happens to be spelt out by die. Another implication is that the term “(generalized) resumptive” is also perhaps not best suited for the use of pleonastic die, in that it does not really “resume” the preceding constituent. It remains thus to be seen whether pleonastic die is intrinsically different from Salvesen's generalized resumptives. Observe that Scandinavian *så* actually has an adverbial origin, which is not the case for die.

The prediction of the Force die 2 analysis is that all constituents which can satisfy the Force-V2 constraint can immediately precede pleonastic die and that constituents that cannot satisfy the V2 constraint cannot immediately precede pleonastic die. Or put differently, *ceteris paribus*, the insertion of pleonastic die should be possible in all V2 sentences in the Ghent dialect.

In the present section, we examine some consequences of our analysis: section 6.1 returns to the co-occurrence of fronted specialized resumptives with pleonastic die introduced in section 4.5; section 6.2 focusses on the prediction that any constituent satisfying the Force-V2 requirement in a V2 sentence should also satisfy the die 2 condition, and, conversely, that a constituent unable to satisfy the Force-V2 condition also does not satisfy the Force die 2 condition.

### 6.1. Co-occurrence with fronted specialized resumptive adverbs

Following the Poletto/Wolfe typology of V2 and the assumption that the Ghent dialect is a Force-V2 language, the CLD pattern with a fronted specialized resumptive adverb illustrated in (38a) is derived as in (38b): the antecedent of the specialized resumptive, the PP *in Sint Kruis* (“in Sint Kruis”), occupies the specifier of the clause-external projection FrameP [cf. (37a)]. The fronted specialized resumptive, here locative *daar* (“there”), occupies the specifier position of ForceP and satisfies the Force-V2 requirement. The finite verb moves to Force, via Fin.


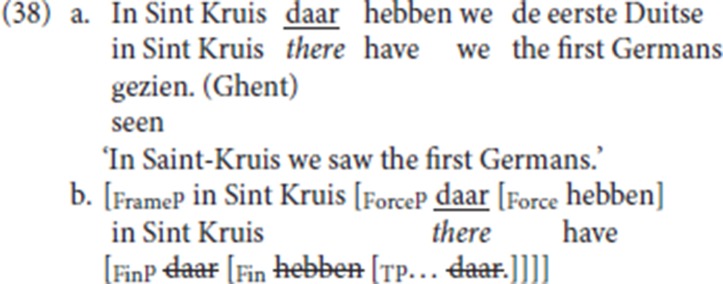


As schematized in (39a), we predict that pleonastic die can co-occur with a fronted specialized resumptive adverb, in effect giving rise to a V4 pattern. The prediction is correct, the relevant pattern was illustrated in (30d), repeated here as (39b), with the partial representation in (39c). The finite verb halts at Fin and die is inserted in Force to satisfy the Force-V2 requirement. The PP *in Sint Kruis* (“in Sint Kruis”), the “antecedent” of the fronted specialized resumptives *daar*, occupies the specifier of the clause-external FrameP; locative *daar* (“there”), the fronted specialized resumptive, occupies the specifier position of ForceP and satisfies the Force die 2 requirement. In this example, pleonastic die is doubled, we assume this is just an effect of repetition after some hesitation.


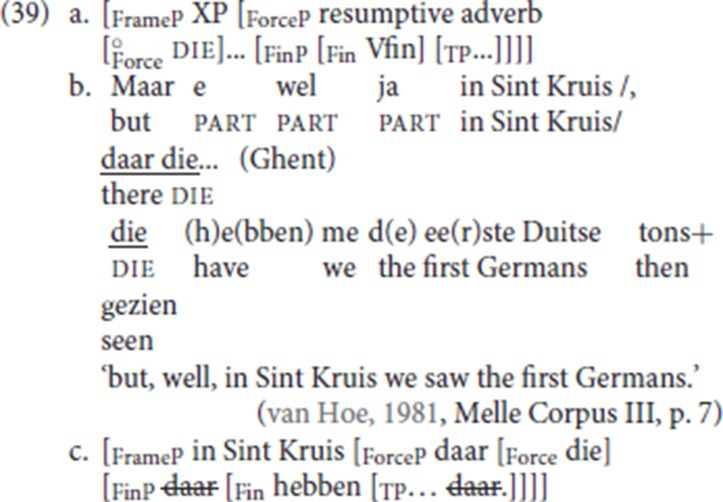


### 6.2. Restrictions on the antecedent

#### 6.2.1. Constituents that (fail to) satisfy V2 and pleonastic *die*

If the constituent to the immediate left of pleonastic die satisfies the Force-V2 requirement, constituents which fail to qualify as the first constituent in a V2 pattern should not qualify as “antecedents” for pleonastic die. Conversely, any constituent that satisfies the Force-V2 constraint should be able to function as the first constituent with pleonastic die. To illustrate this point, we will examine the compatibility of the pleonastic die pattern with the conjunctive adverb *ofwel* (“either”) on the one hand and with the closely similar conjunction *of* (“or”), on the other. We return to a problematic aspect of the second component of the prediction in section 7.

All examples in (40) are intended as a continuation of the first line and they illustrate the uses of *ofwel* (“either”) and of *of* (“or”) as first constituents in the context of V2 patterns. Though intuitively speaking, *ofwel* (“either”) and *of* (“or”) are interpretively similar, they differ in terms of their interaction with V2: for Flemish speakers, the adverb *ofwel* (“either”) satisfies V2 (40a,b)^26^ whilst the conjunction *of* (“or”) does not (40c,d)^27^. Let us tentatively assume this is because *ofwel* is phrasal and *of* is a coordinating head.


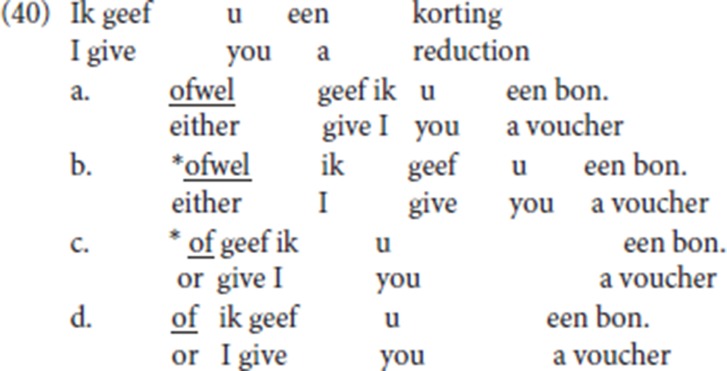


If the constituent immediately preceding pleonastic die simply satisfies a Force-V2 requirement, we predict that *ofwel* will be able to antecede pleonastic die. The data in (41) confirm this prediction: (41a) and (41b) are attested, (41c) and (41d) are based on our informant CM's acceptability judgements.


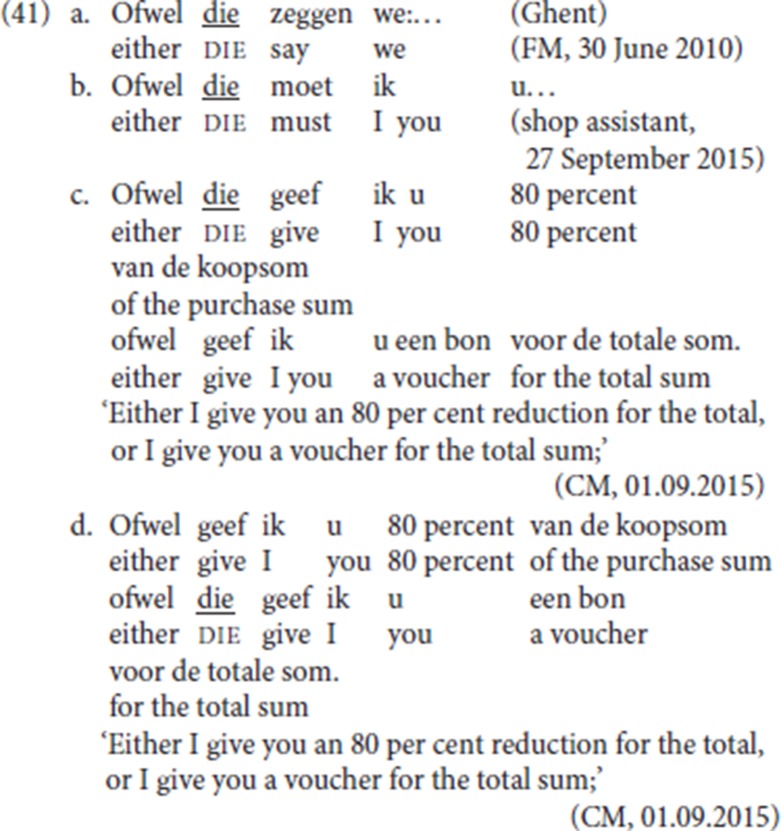


Our account correctly predicts that the conjunction *of* (“or”), by hypothesis a head, cannot constitute the antecedent for pleonastic die: our corpus provides no attestations of the conjunction *of* (“or”) as antecedent of pleonastic die and our informant CM, who accepts pleonastic die after *ofwel* (41)c-d, does not accept pleonastic die after *of* (“or”) (42).


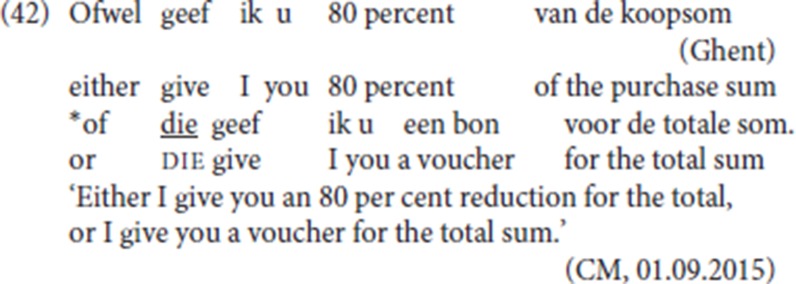


#### 6.2.2. Pleonastic *die* and weak subjects

If the constituent preceding pleonastic die serves to satisfy the V2 condition on Force, any constituent able to satisfy the V2 requirement should be a licit “antecedent” for pleonastic die. So far, we have focused mainly on sentences with initial adjuncts followed by pleonastic die. However, in the course of the discussion, we did include instances with initial *wh*-arguments (section 2.3.2). This in fact suggests we need to take a broader view: examples such as (43) with a nominal initial constituent and which we would have considered as instantiations of CLD in section 3.2.1, see example (17b), could be viewed as further instantiations of pleonastic die:^28^


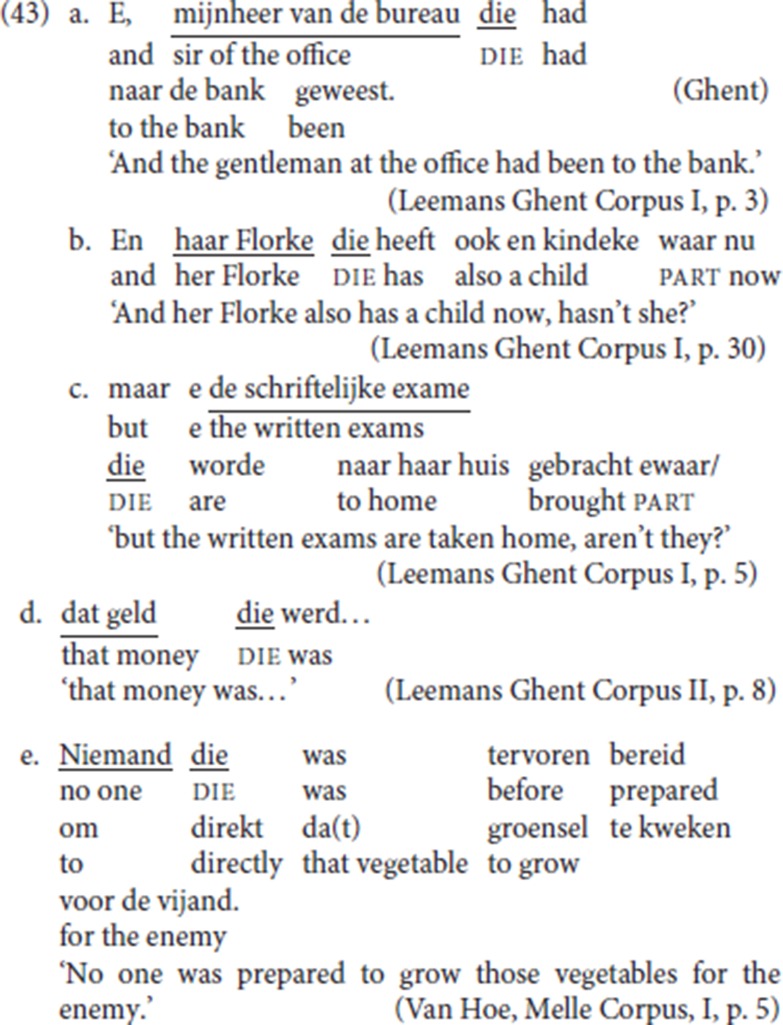


While weak pronouns/clitics can be the initial constituent in V2 patterns, there are no instances in the corpus of pleonastic die following a clitic or weak subject pronoun, and the informant (CM) whom we consulted judged them as ungrammatical both with referential and non-referential weak pronouns.


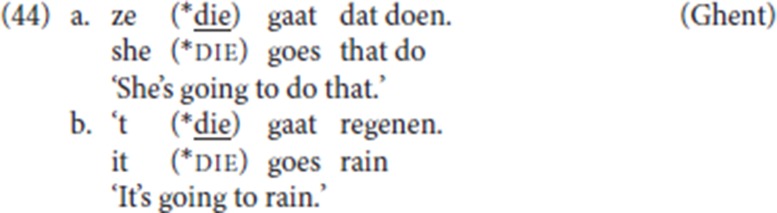


The restriction only concerns weak subject pronouns. Strong subject pronouns, like subject DPs in general, can antecede *die*, as shown in the following example from the corpus:


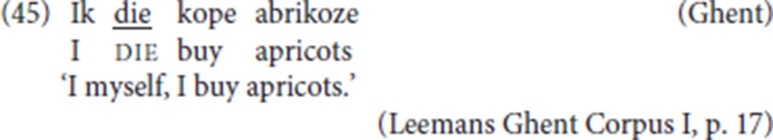


These data need further research. We speculate that the observed restriction is related to the syntax of subject initial V2, and in particular that weak pronouns must be in a spec-head relation with a head carrying agreement features.

## 7. Pleonastic die as a root declarative complementizer

In this section, we look at a number of additional patterns for which our prediction about the suitable antecedents for pleonastic die at first sight seems not to hold and we also refine our analysis of pleonastic die postulating that pleonastic die instantiates a declarative complementiser. This section is rather more speculative, the issues raised here will require further research.

### 7.1. V1, null operators and pleonastic die

If the constituent immediately preceding pleonastic die merely serves to satisfy the V2 requirement on the head Force, any constituent satisfying the V2 requirement in a regular V2 pattern should qualify as “antecedent” for pleonastic die. This prediction faces an empirical problem with respect to *yes/no* questions and imperatives.

It is well known that both *yes/no* questions and imperatives in StD display a linear Verb first (V1) order. (46) contains two relevant examples:


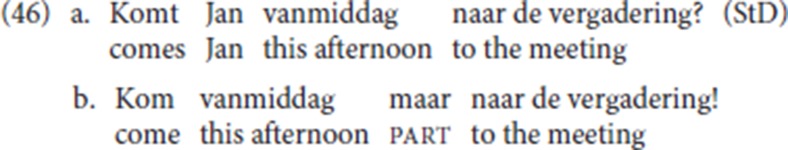


The V1 order in *yes/no* questions and in imperatives is standardly considered compatible with the V2 nature of StD, on the hypothesis that a null operator in sentence-initial position satisfies the V2 condition. In line with our cartographic implementation sketched above and bearing in mind that the null operators would encode interrogative and imperative force respectively, let us assume that in the relevant examples the verb targets Force and that the null operator occupies the specifier of ForceP. We assume that the null operator transits via SpecFinP, giving rise to the bottleneck effect.


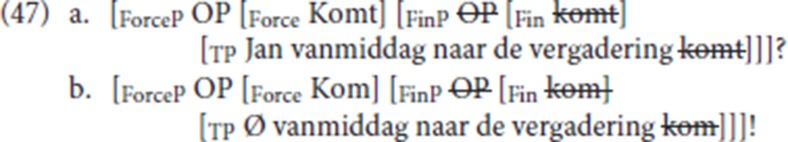


On this scenario, both in imperatives and in *yes/no* questions a null operator satisfies the Force-V2 constraint. All things being equal, then, pleonastic die should also be able to be inserted in *yes/no* questions and in imperatives, effectively leading to a pattern without an overt antecedent^29^. However, there are no attestations of these predicted patterns: we have seen that pleonastic die requires an overt antecedent (section 2). *Yes/no* questions and imperatives are judged by our informant LdG to be incompatible with pleonastic die, (48):


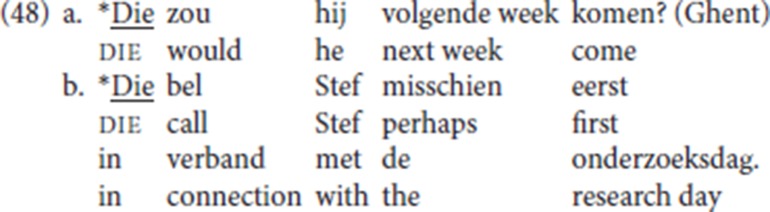


The obvious problem with (48) is that pleonastic die is initial and lacks an overt antecedent, but recall that according to our analysis, die instantiates Force and that its “antecedent requirement” boils down to the Force-V2 requirement. So, in formal terms, our analysis no longer predicts that (48) should be ungrammatical: if a null operator can satisfy the V2 requirement in a regular *yes/no* question and in an imperative, with the verb in Force to satisfy the Force-V2 requirement, then it should do so too in (48) when Force is realized as die. If a first constituent is added to the illicit patterns, pleonastic die remains incompatible with *yes/no* questions or imperatives. We tested the following with our informant LdG, who was adamant that these were all unacceptable: (49a-d) illustrate *yes/no* questions, (49e) an imperative:


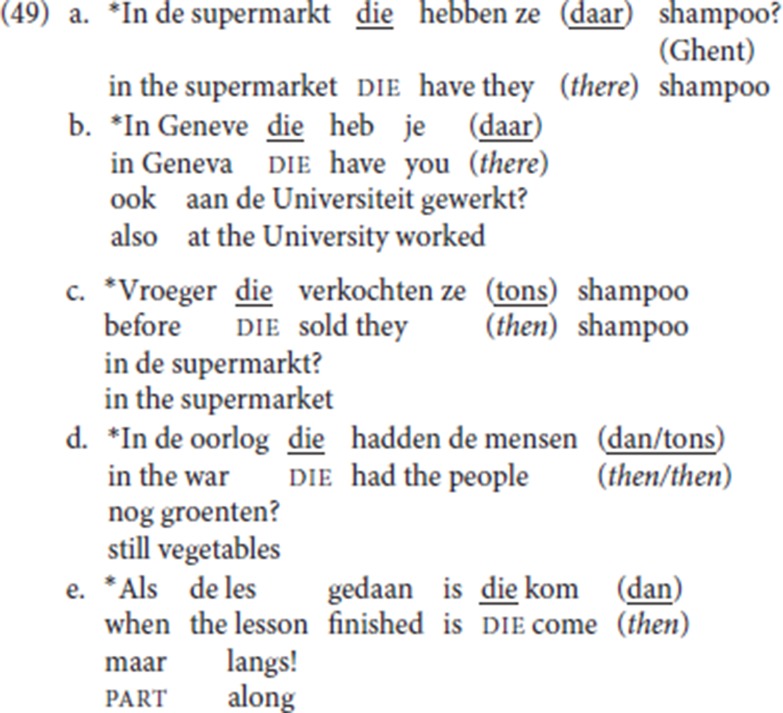


We analyze pleonastic die as a root complementizer. The data in (48)–(49) show that not all root clauses in the Ghent dialect admit the insertion of the root complementizer die. This is not unexpected; after all, the insertion of the complementizer is sensitive to the features of Force. To give a straightforward example: English *if* and *whether* typically go with interrogatives while *that* is used with declaratives (though there is important speaker variation in the use of *that*, see Radford, 2018).

As a first stab, let us refine our analysis and propose that pleonastic die is a declarative root complementizer. It immediately follows that it will serve to introduce statements and that it will be incompatible with non-declarative clauses such as *yes/no* questions and imperatives^30^.

An immediate problem is of course that pleonastic die is compatible with *wh*-questions as shown by the elicited data in (13) repeated in (50). Though not all speakers accept the pattern, six out of 12 speakers rate (50a) with a score of 4 or 5 and five out of 12 speakers rated (50b) with a score of 4 or 5 (cf. section 2.3 and footnote 10).


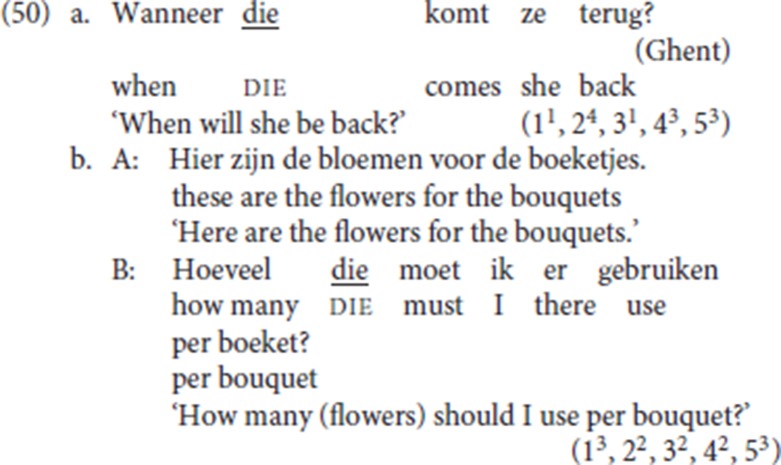


In (50), pleonastic die occurs in what amounts to a question. But note that the questions concerned are constituent questions, i.e., questions presupposing the truth of the associated proposition: (50a) presupposes that “she is coming back,” (50b) was explicitly set in a context in which the speaker will be using the flowers. We speculate that the acceptability of pleonastic die in such examples is due precisely to the fact that the clausal constituent associated with the initial *wh*-phrase is presupposed. The analysis entails that we cannot view pleonastic die as the spell out of assertive illocutionary force, because in the case of *wh*-questions the clausal complement of pleonastic die is presupposed, rather than asserted. In order to capture these data we would have to resort to an approach according to which “declarative” is negatively defined as the default value of clause typing for clauses that are neither *yes/no* questions nor imperatives^31^. This speculation clearly requires more work; the type of approach to “declarative” that we envisage is found, for instance, in Roberts and Roussou (2002, p. 141) who say:

Instead of saying that we have a C [+ declarative], we have C = declarative by default, where no subfeature is present, and C = Q, Exclamative, and so on, as marked subfeatures.

Such an approach would be in line with the fact that, for instance, complements of factive verbs or finite temporal adverbial clauses are also “declarative,” though they do not constitute assertions. For some discussion of the latter clause types, see Haegeman and Ürögdi, 2010a,b) and the references cited.

Possibly, though at this point this remains a mere speculation, the insertion of pleonastic die in *wh*-questions may in fact highlight or reinforce the presuppositional effect on the complement of the *wh*-phrase^32^^/^^33^.

### 7.2. Pleonastic die as a declarative complementizer: the *dat/die* alternation

If pleonastic die is inserted as a declarative complementizer in Force, the question arises why the complementizer takes the form *die* and why it is not possible to insert the regular declarative complementizer *dat*, the regular complementizer in the Ghent dialect, already illustrated in (15d) above.

Schematically the data are summarized in (51a); examples such as (51b) and (51c), with *dat* instead of die, are unattested and are judged unacceptable by our informants.


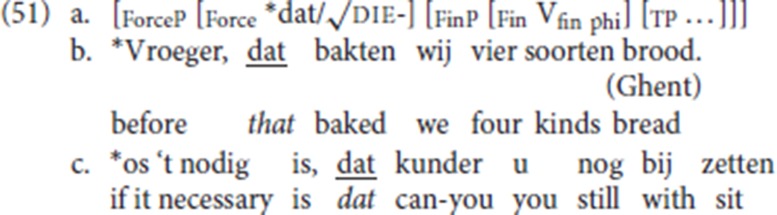


An alternation between the formatives *dat* and *die* is not completely novel. We might in fact interpret the form *die* of the pleonastic element as an alternative realization of the declarative complementizer *dat* which is a byproduct of the proposed derivation of pleonastic die sentences. Schematically, this would mean that the underlying form of pleonastic die is the regular complementizer *dat* and that for some reason, which will be clarified below, this formative has to be converted to die^34^. We continue to assume that the initial constituent in the pleonastic die sentence (52a), here *morgen* (“tomorrow”), first satisfies the V2 constraint on Fin and that it moves from SpecFinP to SpecForceP, leaving a copy in SpecFinP. Given this assumption and considering that copies correspond to traces in the earlier incarnation of our theoretical model, (52a) has the notational variant (52b), which instantiates a sequence of the complementizer *dat* followed by a trace. Configurationally (52)b can be viewed as an instantiation of a violation of the *that*-trace filter (Chomsky and Lasnik, 1977), arising through the movement of the constituent from SpecFinP to SpecForceP across the declarative complementizer *dat*.


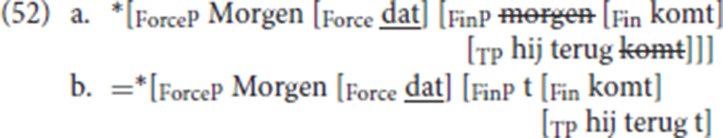


Originally, the *that*-trace filter (Chomsky and Lasnik, 1977) was formulated to handle the ban on subject extraction in English examples such as (53a): extracting the *wh*-subject *who* leads to ungrammaticality. In the grammatical variant (53b) *that* is deleted. On the other hand, object extraction is not sensitive to the presence of *that*, as shown in (54), in which the *wh*-object can be extracted regardless of the presence of the complementizer *that*.


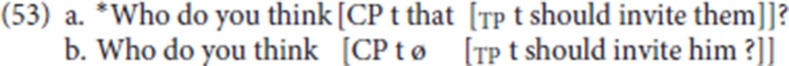



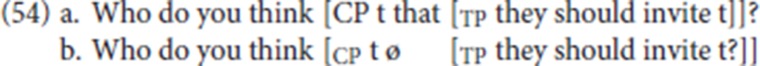


It is well known that some *that* trace violations are “repaired” by a morphological change in the complementizer. English (53) is one case in point: replacing *that* by its null alternative (ø) rescues the pattern. We will not dwell on this here (see Rizzi and Shlonsky, 2006, 2007 for a recent analysis). Another well known example of this type of morphological repair is illustrated by French (55): whereas direct object *que* (“what”) can be successfully extracted across the complementizer *que* (“that”) (55a), extraction of subject *qui* (“who”) from its canonical position across the adjacent complementizer *que* leads to ungrammaticality: (55b) violates the *that*-trace filter. (55b) can be repaired by substituting the form *qui*, which has “nominal” properties (Rizzi and Shlonsky, 2006, 2007), for the regular complementizer *que* (55c), thus replacing the offending sequence *que-*trace by the licit *qui-*trace.


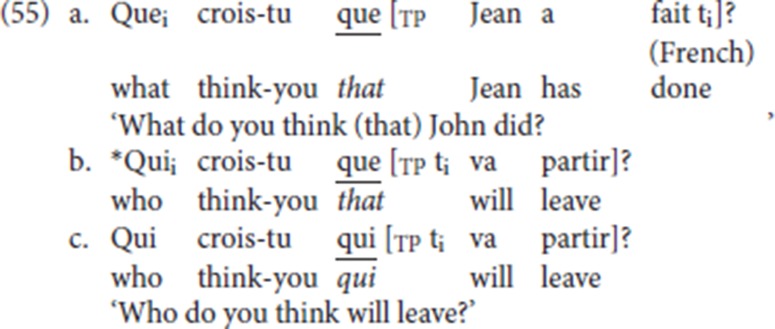


Our earlier (52b) also instantiates a *that* trace sequence, though, of course, the trace here is not a subject trace. Just like replacing *que* by “nominal” *qui* alleviates the *that* trace effect in French (55c), replacing the formative *dat* by *die*, can be taken to repair the *dat-*trace violation. (56) is a first tentative representation. Obviously, viewing the obligatory spell out of Force by die as a reflex of the *que/qui* alternation will require further work, in particular in relation to current views on the nature of the *que/qui* alternation (cf. Rizzi and Shlonsky, 2006, 2007)^35^.


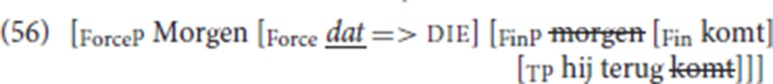


### 7.3. Ellipsis and the phatic use of pleonastic die

One issue that we have not addressed so far concerns the motivation for the insertion of the root declarative complementizer die. Tentatively we have associated pleonastic die with a “declarative” value, but so far, the insertion of pleonastic die seems completely optional and does not add to the interpretation of the sentence, which is why we used the term “pleonastic.” This complete optionality is rather unexpected: true optionality runs counter to economy principles. However, an extension of the data suggests that pleonastic die may have some discourse related interpretive function. This section is speculative.

(57) illustrates attestations of ellipsis of the complement of pleonastic die: the fact that a longer form of die is chosen can be related to the need to license the ellipsis. The elliptical patterns are quite common and they seem to be used by speakers to hold the floor while further elaborating their contribution to the conversation.


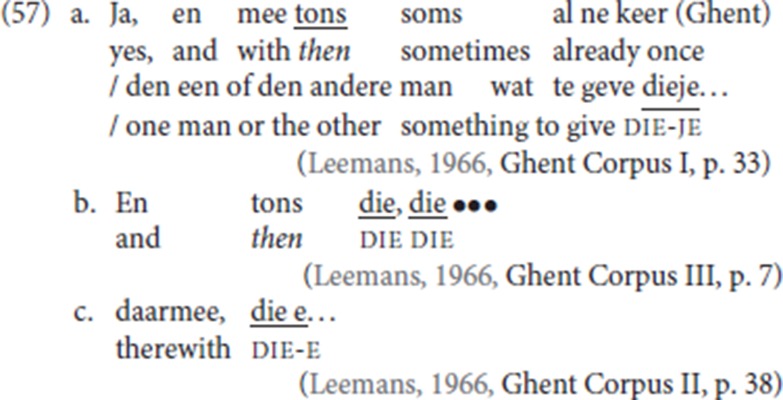


In addition, pleonastic die is also used in isolation, i.e., with ellipsis of both its complement and the “antecedent.” With respect to this isolated use, it has been noted (Luc De Grauwe p.c., also anecdotal observations by Liliane Haegeman) that speakers use the pattern as a conversational move with a purely phatic function. Luc De Grauwe (p.c., email) reports:

“bij ontmoetingen (met mezelf of in ruimer familieverband) viel soms een keertje een korte stilte in het gesprek/ de small talk. Dan had mijn tante de gewoonte, telkens als eerste die stilte te doorbreken door het uitspreken van het enkele woordje *dieë* (met langgerekte eerste lettergreep)—dit gewoon om het gesprek weer op gang te (laten) brengen, eventueel met een ander onderwerp.”

Translation (kdc-lh)

*in the course of meetings (with myself or in the larger family circle) it would happen that a sudden silence occurred in the conversation/small talk. Then my aunt had the habit to be the first to interrupt that silence by pronouncing the word* dieë *(with long first syllable)—with the sole purpose of getting the conversation going again (possibly on a different topic) (Luc De Grauwe, pc, 16.08.2017, email)*

If pleonastic die spells out a “declarative” root Force head, the use of the declarative complementizer die in isolation could be seen as a conversational move by which the speaker “declares” his intention to speak by this minimal illocutionary act and thus takes and/or holds the floor. The use of pleonastic die would then be the overt encoding of the speaker's commitment to a speech act, be it as a way of taking the floor or continuing to hold the floor, and thus to signal that the speaker continues to be engaged in the communictative exchange. Pleonastic die would also signal that the intended speech act will not be a *yes/no* question or an imperative.

## 8. Summary: force-V2 and pleonastic die

This paper discusses the use of the pleonastic particle die in the Ghent dialect. The particle is used in a V3 pattern in which the first constituent is an adverbial adjunct, followed by the particle die, followed by the finite verb.

Though, at first sight, pleonastic die could be taken to be a generalized counterpart to the fronted specialized resumptive adverbs *dan* (“then”), *daar* (“there”), etc. in the adverbial CLD pattern, and which are also available in the dialect under consideration, there are a number of arguments for not assimilating the two patterns. We propose that while the fronted specialized adverbs are phrasal operators moved to the left periphery, pleonastic die is a head directly merged in a left-peripheral position.

In terms of the Poletto/Wolfe typology, the pleonastic die pattern in the Ghent dialect is argued to instantiate a variant of the Force-V2 pattern: pleonastic die is inserted in Force, which requires an obligatory specifier to satisfy the “V2 condition.” Exotic though they might seem at first, the Ghent pleonastic die sentences are thus argued to be a twist on the Force-V2 implementation.

It is proposed that pleonastic die is a root complementizer which is inserted in declarative clauses.

Placed in a broader perspective, our paper provides evidence for micro-variation in the syntax of V2, and it also highlights the fact that what seem like superficial V3 patterns do not necessarily receive a uniform analysis. If Ghent pleonastic die can be categorized as a generalized resumptive, then our paper also shows that while at first sight near equivalent, generalized resumptive constituents and specialized resumptive constituents may not necessarily have the same syntax.

## Author contributions

KD contributed sections 1–3 and 7. LH contributed sections 4–6.

### Conflict of interest statement

The authors declare that the research was conducted in the absence of any commercial or financial relationships that could be construed as a potential conflict of interest.

## Footnotes

^2^As pointed out by Malte Zimmerman, our discussion of the semantic matching between initial constituent and adverbial remains relatively informal and vague. There are indeed a number of issues that arise, such as the fact that the same adverb functions as a resumptive for a conditional clause and a temporal clause. Though the conditions on the matching of specialized resumptives are of interest and may shed light on the way generalized resumptives arise diachronically, we will not go into it here because the focus of our paper, the Ghent resumptive element die, is typically not matched with its antecedent. With respect to the fact that what seems a specialized resumptive matches both temporal and conditional adverbials, we tentatively follow a suggestion by this reviewer and assume provisionally that the temporal/conditional adverbials resume topic situations, which can be introduced by topical conditional clauses or by temporal clauses (see also Salfner and Salfner, 2011 for more thorough discussion). However, the data put forward in (9) and section 4.7 show that a topic analysis for all data is untenable.

^3^Many Flemish speakers systematically use *dan* for both past and future/conditionals. This correlates with the fact that, for those speakers, temporal clauses related to the past are not introducted by the conjunction *toen* (“when_past_”), but rather by *als* (“if, when”), which can thus be used for both past adverbial clause modifiers and for future/conditionals.

^4^We use the term ‘particle’ pre-theoretically.

^5^Vanacker (1980, pp. 77–78) suggests that pleonastic die may have originated as the instrumental use of the demonstrative. To the best of our knowledge, there are no records that trace the diachronic development of the pattern.

^6^De Clercq and Haegeman (2017) point out one exception. For reasons of space we cannot go into this here.

^7^12 informants from Ghent participated in our survey. We have only selected speakers who confirmed that they were users of the die pattern. Each informant rated 52 sentences containing die on a 5-point Likert scale, with 1 being unacceptable and 5 being fully acceptable. The sentences aimed at testing which type of antecedent die can combine with, whether die can also appear in midposition and hence whether two occurrences of die can co-occur in one clause, whether it can appear without antecedent and can combine with resumptive *dan* “then” in a clause. For every test sentence that we use we will report how many of our informants gave a particular score. For instance, 1^9^ means that 9 informants considered the sentence unacceptable and gave it the score 1. If informants gave 3, 4, or 5, we considered the sentence acceptable. Twenty-one representative sentences of the survey are provided in the Appendix (Table 1 in Appendix). In order to illustrate intra- and interspeaker variation, the appendix reports on the results per speaker for a selection of test sentences. More in particular, this information is provided for those sentences which contain a selected PP (Table 2 in Appendix) and a *wh*-word (Table 3 in Appendix), cf. footnotes 9 and 10.

^8^See the appendix for an overview of speaker variation with respect to the data in (11)a-b-c. Table 2 in the Appendix shows that whereas there is variation amongst speakers as to which type of antecedent for die they allow, all (apart from one) speakers are unanimous when it comes to rejecting sentences with die in midposition. Consequently, the contrast between fully ungrammatical sentences (those with die in midposition) and sentences that display interspeaker variation, like those in (11) is very clear.

^9^See the appendix for an overview of inter- and intraspeaker variation with respect to the sentences in the survey that contain *wh*-words. Table 3 in Appendix shows that there is variation amongst speakers as to which type of *wh*-antecedent for die they allow. However, in general, we can say that a substantial part of the speakers accept some *wh*-words followed by die and two informants accept most *wh*-words followed by die. As signaled in note 9, these acceptability ratings for sentences with *wh*-words differ substantantially from the ratings for fully unacceptable sentences like those with die in midposition (Table 3 in Appendix, sentence Q8), which speakers reject unanimously. This observation is important for the argumentation developed in section 4, because it supports the idea that an analysis of die along the lines of adverbial CLD is not tenable.

^10^For completeness'sake, the counterpart with a specialized resumptive in Dutch is given in (i) Reconstruction is available in the non resumptive pattern as well as with resumptive *dan*. This is in line with Haegeman and Greco (2018) analysis: for (i) it can be plausibly argued that the specialized *dan* has moved from a clause medial position.

^11^Like other Flemish dialects the Ghent dialect uses what used to be the accusative form of determiner elements also for nominatives. When a demonstrative is used without the nominal that it modifies, a strong form is used and this is preceded by a determiner. This reduplication pattern also arises with possessors (see Haegeman, 2004, pp. 218–219; Haegeman, 2013, p. 244). So in (ia) the possessor *menen* (“my”) is used in front of the nominal *auto* (“car”). In (ib), the nominal is omitted and we find a strong form of the possessor *mijnen* (“my”) preceded by the definite article *de* (‘the’). For reasons of space we do not go into this here. Observe that the strong demonstrative patterns *den dienen/de die* differ from the subject doubling patterns described in Haegeman (1990) for West Flemish and illustrated in (ii), which should be compared with (iii). The sequence *ze zie* in (ii) consists of a weak pronominal form *ze* followed by a strong form *zie*. But, as shown in (iib) and (iic), the two elements may be separated: in (iib), an object clitic intervenes between *ze* and *zie*, and in (iic), *ze* has moved to initial position in a V2 configuration. As shown by (iid) and (iie), *ze zie* cannot be used as a non-subject. The patterns contrast with the strong demonstrative (iiia), whose components cannot be separated (iiib,c) and which can have non-subject functions (iiid,e). For a first formal analysis of subject doubling in West Flemish see Haegeman (1990); for an application to the Ghent dialect see de Geest (1990).

^12^In section 5.2 we account for the absence of complementizer agreement on pleonastic die. Section 7.3 will show that the particle is not completely invariant.

^13^Alternatives with *dat* are attested and judged acceptable. In a separate acceptability judgement test, one informant graded (i) with *die* a score 6/7 and indicated that *die* could be replaced by *dat*. The pattern in which the initial constituent is picked up by *dat* may in fact be an instantiation of the pattern in (17), i.e. with *dat* the neuter analog of *den dienen* [cf. (17a)].

^14^Hoekstra (1999, p. 66) gives (i) as ungrammatical. See also Broekhuis and Corver (2016, p. 1458). In the Ghent dialect resumptive *den dienen* cannot take a bare quantifier as its antecedent (C.M. 04.032018, p.c): This suggests that the resumptive pattern with *den dienen* is closer to the StD CLD pattern. We leave this observation for future research.

^15^Zwart (1997, pp. 249–250) also suggests in a footnote that pleonastic die is the adverbial variant of the resumptive *die* used in topicalization. He does not link it to other instances of adverbial resumption.

^16^In contrast, when *die* resumes an argument nominal in CLD, mid position is available. This pattern is like the StD CLD pattern [cf. Den Dikken and Surányi, 2017, p. 551, his (14c)] The resumptive demonstrative *die* remains *in situ* in (ib) because the left-peripheral slot which it would target is occupied by *waar* (“where”). Following Mikkelsen (2015), we assume that its discourse feature can be licensed through the left-peripheral operator *waar*. See her paper for more details. In (ic) *die* is the referentially used demonstrative which alternates with *den dienen* (“that”). It is a phrasal constituent. Again it cannot move to the left periphery because in imperatives the left-peripheral slot is activated. See Mikkelsen (2015) for similar patterns. We intend to look into the Ghent CLD pattern with argument nominals and the alternation between *die* and *den dienen* in future work.

^17^Thanks to Malte Zimmerman for bringing this point to our attention.

^18^In this respect they again pattern with the demonstrative pronominals that function as resumptives in the CLD pattern.

^19^Judgements may vary: notably one of the authors of this paper, Karen De Clercq, has divergent judgements in particular with respect to the distribution of *dan* (“then”). We hope to return to this point later.

^20^German *da* patterns differently here. Katharina Hartmann (p.c.) gives the following judgements: while in (i) *in situ dann* (“then”) is judged as slightly better than *in situ* da, the latter is still “very good.” In (ii) she grades both options as equally grammatical.

^21^Katharina Hartmann (p.c.) points out that German *da* is available in the following. The pattern in (i) differs from that in (32) in that *da* is here accompanied by an *in situ* resumptive *dran* (“thereon”). Th equivalent of (i) with pleonastic die would be ungrammatical in the Ghent dialect.

^22^For expository reasons, we cannot develop the point in detail here but one typical property of constituents in FrameP which give rise to a V3 order in West Flemish is their inability to reconstruct, this in contrast with the initial constituent in a “regular” V2 root clause (cf. section 2.4). In West Flemish (ia) below, the initial constituent *drie moanden no datum* (“three months later”) in the regular V2 clause may be taken to modify either the time of the intention or the actual time of the wedding. In (ib), on the other hand, the same modifier is clause-external, by hypothesis in Haegeman and Greco's FrameP, and it can only modify the time of the intention:

^23^External merge of another constituent in the CP area is also unavailable, because such a constituent would itself block movement of the constituent in SpecFinP to SpecForceP. Such an externally merged constituent by hypothesis satisfies a criterial feature of a functional head in the CP area and cannot itself move to SpecForce.

^24^One option that does not seem to be ruled out by the Poletto/Wolfe V2 languages is that in which the specifier of FinP is filled by one constituent, the finite verb moves to Fin and then to Force, and another constituent is merged in SpecForceP, which would be like the mirror image of our analysis of pleonastic die in which it is the finite verb that remains in Fin and pleonastic die that is merged in Force. We have nothing to say about this issue here and hope it can be clarified in future work.

^25^Haegeman and Greco (2018) postulate that there is micro-variation between West Flemish and StD in relation to subject initial V2. Interestingly, the core difference which they postulate also hinges on the landing site of the finite verb in the left periphery. We refer to their paper for details and for additional references.

^26^The Netherlandic and Belgian varieties of Dutch differ: Netherlandic varieties allow for both (ia) and (ib), though (ib) is the majority choice; in Belgian varieties of Dutch (ia) is the form used (Electronic ANS: http://ans.ruhosting.nl/e-ans/25/05/02/body.html). We have nothing to say about this variation here.

^27^Either *ofwel* is merged in SpecFinP and moves to SpecForceP or, alternatively, it actually is merged in a TP-internal position (cf. Larson, 1985; Schwarz, 1999; Den Dikken, 2006 on *either*).

^28^Thanks to Katharina Hartmann for raising this point.

^29^Thanks to Giuseppe Samo for bringing up this point.

^30^Thanks to Luc de Grauwe for very helpful discussion of these examples.

^31^Malte Zimmerman suggests that we could also investigate whether the insertion of *die* in *wh*-clauses is dependent on whether the addressee is knowledgable with respect to the question content. In this way we might be able to avoid the use of default features in the analysis and we could link the import of Force to the addressee, in line with work by Gunlogson (2003), who shows that declarative yes/no-questions presuppose the addressee to be informed on the question content. The idea would be that the *wh*-questions with *die* also presuppose the addressee to be informed on the question content. Unfortunately, a preliminary investigation did not lead to any clear results on this: the informant we consulted detected no difference in acceptability of die in *wh*-questions in relation to the knowlegeability of the adressee. More research will be required to determine the precise conditions regulating the availability of die with *wh*-questions.

^32^This point needs to be further pursued. In particular we speculate that the clause typing effected by pleonastic die is like that which leads to Referential clauses in the sense of Haegeman and Ürögdi (2010a,b). However, for reasons of space we cannot elaborate this here.

^33^Consider also the attested (i). Our own informant CM explicitly points out that she rejects this type of *die dat* sequences, so there is clearly variation across speakers. For speakers accepting (i) the position of *die* would be compatible with our proposal that pleonastic die spells out a declarative Force head. In the embedded clause, *die* precedes the regular (inflected) complementizer *da*: if we assume that the latter occupies Fin, then *die* could be in Force, where it would alternate with *of* (‘if’) for interrogatives:

^34^There are of course other cases in which clauses introduced by *dat* can function as independent clauses: exclamatives such as (i) are a case in point. But in these patterns the finite verb remains in its TP-internal position: In these examples the conditions for converting *dat* to *die* are not present.

^35^Observe that while we do not attribute any specific interpretive property to the *dat/* die alternation here, it remains true that the rescue strategy summarized in (56) might be taken to add a nominal flavor to the neutral filler *dat* for Force. We also need to explore to what extent the nominal nature of pleonastic die can be related to the referential status of the clause in the sense of Haegeman and Ürögdi (2010a,b).
