# Supplementary material for: The Typology of V2 and the Distribution of Pleonastic die in the Ghent Dialect
Source: Front Psychol. 2018 Aug 29;9:1342. doi: 10.3389/fpsyg.2018.01342 (PMC6123374; doi:10.3389/fpsyg.2018.01342)
Supplement: Supplementary file 1 [file Image_1.pdf]

Appendix

Table 1 DIE-survey of sentences relevant for the paper; 5-point Likert scale; frequencies, with glosses and idiomatic translations.

		1	2	3	4	5
		😫	🙁	😐	🙂	😃
1.	A: Jef komt morgen voor de katten zorgen.<br>A: ‘Jef will take care of the cats tomorrow.’<br><br>B: Die kunnen we met een gerust hart<br>*B: DIE we can we with a quite heart*<br>naar de cinema gaan.<br>*to the movies go*<br><br>B: ‘So we can go to the pictures without worrying’.	9	2	0	0	1
2.	Waarschijnlijk die is hij weeral ziek.<br>*probably DIE is he sick again*<br><br>‘Probably he is sick again.’	2	1	2	5	2
3.	In mijn stoverij die doe ik nooit peperkoek.<br>*in my stew* DIE *put I never gingerbread*<br><br>‘I never add gingerbread to my stew.’	1	1	4	3	3
4.	Op ‘t derde verdiep die zou ik niet willen wonen.<br>*on the third floor DIE would I not want live*<br><br>‘I would not want to live on the third floor.’	1	1	3	4	3
5.	In 1954 die is hij geboren.<br>*in 1954 DIE is he born*<br><br>‘he was born in 1954.’	3	1	4	1	3
6.	Wanneer die komt ze terug?<br>*when DIE comes she back*<br><br>‘When is she coming back?’	1	4	1	3	3
7.	A: Hier zijn de bloemen voor de boeketjes.<br>A: ‘Here are the flowers for the bouquets.’<br><br>B: Hoeveel die moet ik er gebruiken per boeket?<br>*B: how many DIE must I there use per bouquet?*<br><br>‘How many do I use per bouquet?’	3	2	2	2	3
8.	Als het regent, wat gaan we die doen?<br>*if it rains, what go we DIE do?*<br><br>‘If it rains, what are we going to do?’	11	1	0	0	0
9.	Als het regent, wat die gaan we doen?<br>*if it rains, what DIE go we do*	9	2	0	1	0

	‘If it rains, what are we going to do?’					
10.	Als het regent, zelfs die ga ik te voet naar het werk.<br>*if it rains, even DIE go I on foot to work*<br><br>‘When it rains, even then I walk to work.’	8	3	1	0	0
11.	Toen de bel ging, juist die ging ik vertrekken.<br>*when the bell rang, just DIE went I leave*<br><br>‘When the bell rang, just then I was about to leave.’	8	2	1	0	0
12.	A: Wanneer komt ze terug?<br>A: ‘When is she coming back?’<br><br>B: Volgende vrijdag die komt ze terug.<br>*B: next Friday DIE comes she back*<br><br>B: ‘She is coming back next Friday.’	1	0	5	1	5
13.	A: ‘t Is mijn verjaardag. Ik wil een feest geven.<br>A: ‘It’s my birthday. I want to give a party.’<br><br>B: Wie die wilt ge allemaal inviteren?<br>*B: who DIE want you all invite*<br><br>B: ‘Who do you want to invite?’	6	2	1	0	2
14.	A: ‘t Is mijn verjaardag. Ik wil een feest geven.<br>A: ‘It’s my birthday. I want to give a party.’<br><br>B: Wie wilt ge die allemaal inviteren?<br>*B: who want you DIE all invite?*<br><br>B: ‘Who do you want to invite?’	10	1	0	0	1
15.	A: ‘t Is mijn verjaardag. Ik wil een feest geven.<br>A: ‘It’s my birthday. I want to give a party.’<br><br>B: Wie die wilt ge dan allemaal inviteren?<br>*B: who DIE want you dan all invite?*<br><br>B: ‘Who do you want to invite then?’	0	1	4	4	3
16.	De tafel is verzet.<br>'The table has been moved.’<br><br>Waar die moet ik mijn gerief nu leggen?<br>*where DIE must I my stuff now put*<br><br>‘Where can I leave my things now?’	7	2	0	1	2
17.	Vandaag die heeft hij nog een vergadering.<br>*today DIE has he another meeting*<br><br>‘He has another meeting today.’	0	1	4	4	3

18.	Vandaag heeft hij die nog een vergadering.<br>*today has he* DIE *another meeting.*<br><br>'He has another meeting today.'	11	0	0	0	0
19.	A: De serveuse is daar echt niet vriendelijk.<br>A: 'The waitress there is really not kind.'<br><br>B: Daarom ga ik daar die niet graag.<br>*B: therefore go I there not gladly*<br><br>B: 'That's why I don't like going there.'	12	0	0	0	0
20.	A: De serveuse is daar echt niet vriendelijk.<br>A: 'The waitress there is really not kind.'<br><br>B: Daarom die ga ik daar niet graag.<br>*B: therefore* DIE *go I there not gladly*<br><br>B: 'That's why I don't like going there.'	0	2	2	5	3
21	In mijn stoverij doe ik die nooit peperkoek.<br>*in my stew put I* DIE *never gingerbread*<br><br>'I never add gingerbread to my stew.'	11	1	0	0	0

Table 2. Speaker variation for sentences with selected PP antecedents

	Q_4	Q_5	Q_3	Q_21
I1	5	5	5	1
I2	4	3	2	1
I3	3	3	3	1
I4	4	4	4	1
I5	5	5	5	1
I6	2	3	3	2
I7	4	1	1	1
I8	4	1	4	1
I9	1	1	3	1
I10	3	2	3	1
I11	3	3	4	1
I12	5	5	5	1

Table 3. Speaker variation for sentences with wh-antecedents

	wanneer die	*hoeveel die*	*waar die*	*wie die*	*wie die…dan*	*wat…die*	*wat die*
	Q_6	Q_7	Q_16	Q_13	Q_15	Q_8	Q_9
I1	2	1	1	1	1	1	1
I2	3	3	1	2	2	1	1
I3	2	3	1	1	1	1	1
I4	5	5	5	5	4	1	4
I5	5	5	1	1	1	1	1
I6	2	2	2	0	2	2	2
I7	4	4	1	1	0	1	1
I8	4	1	2	2	3	1	1
I9	1	1	1	1	1	1	1
I10	2	2	1	1	1	1	1
I11	4	4	4	3	3	1	1
I12	5	5	5	5	5	1	2
